# Person‐centred interventions to improve patient−provider relationships for HIV services in low‐ and middle‐income countries: a systematic review

**DOI:** 10.1002/jia2.26258

**Published:** 2024-05-13

**Authors:** Laura K. Beres, Ashley Underwood, Noelle Le Tourneau, Christopher Galloway Kemp, Gauri Kore, Lauren Yaeger, Jingjia Li, Alec Aaron, Claire Keene, Deepthi Priyanka Mallela, Banda A. A. Khalifa, Aaloke Mody, Sheree Renae Schwartz, Stefan Baral, Chanda Mwamba, Kombatende Sikombe, Ingrid Eshun‐Wilson, Elvin H. Geng, Marie‐Claude C. Lavoie

**Affiliations:** ^1^ Johns Hopkins Bloomberg School of Public Health Baltimore Maryland USA; ^2^ Centre for Infectious Disease Research in Zambia (CIDRZ) Lusaka Zambia; ^3^ Washington University in St. Louis School of Medicine St Louis Missouri USA; ^4^ Oxford University Oxford UK; ^5^ Center for International Health Education and Biosecurity University of Maryland School of Medicine Baltimore Maryland USA; ^6^ Institute of Human Virology University of Maryland School of Medicine Baltimore Maryland USA

**Keywords:** healthcare delivery, HIV treatment, interventions, patient−provider interactions, person‐centred, systematic review

## Abstract

**Introduction:**

Person‐centred care (PCC) has been recognized as a critical element in delivering quality and responsive health services. The patient−provider relationship, conceptualized at the core of PCC in multiple models, remains largely unexamined in HIV care. We conducted a systematic review to better understand the types of PCC interventions implemented to improve patient−provider interactions and how these interventions have improved HIV care continuum outcomes and person‐reported outcomes (PROs) among people living with HIV in low‐ and middle‐income countries.

**Methods:**

We searched databases, conference proceedings and conducted manual targeted searches to identify randomized trials and observational studies published up to January 2023. The PCC search terms were guided by the Integrative Model of Patient‐Centeredness by Scholl. We included person‐centred interventions aiming to enhance the patient−provider interactions. We included HIV care continuum outcomes and PROs.

**Results:**

We included 28 unique studies: 18 (64.3%) were quantitative, eight (28.6.%) were mixed methods and two (7.1%) were qualitative. Within PCC patient−provider interventions, we inductively identified five categories of PCC interventions: (1) providing friendly and welcoming services; (2) patient empowerment and improved communication skills (e.g. supporting patient‐led skills such as health literacy and approaches when communicating with a provider); (3) improved individualized counselling and patient‐centred communication (e.g. supporting provider skills such as training on motivational interviewing); (4) audit and feedback; and (5) provider sensitisation to patient experiences and identities. Among the included studies with a comparison arm and effect size reported, 62.5% reported a significant positive effect of the intervention on at least one HIV care continuum outcome, and 100% reported a positive effect of the intervention on at least one of the included PROs.

**Discussion:**

Among published HIV PCC interventions, there is heterogeneity in the components of PCC addressed, the actors involved and the expected outcomes. While results are also heterogeneous across clinical and PROs, there is more evidence for significant improvement in PROs. Further research is necessary to better understand the clinical implications of PCC, with fewer studies measuring linkage or long‐term retention or viral suppression.

**Conclusions:**

Improved understanding of PCC domains, mechanisms and consistency of measurement will advance PCC research and implementation.

## 1 INTRODUCTION

Person‐centred care (PCC) offers principles that promise to improve HIV prevention and care, potentially even in resource‐constrained and public health settings that typically emphasize standardization and scale. The initial phases of global HIV response from the first decade after 2005 PEPFAR's initiation appropriately prioritized simplicity, standardization and scale [1] (e.g. the four S's), over time it became clear that services unresponsive to individual circumstances—even when free of charge—are not always able to adequately engage patients and deliver sustained population health gains. In response, the global public health community has increasingly embraced ideas about patient, person or client‐centredness as a priority for high‐quality, responsive health services [2, 3, 4, 5]. For example, Differential Service Delivery (DSD) Models that seek to modify the nature, frequency and location of services to minimize unnecessary burdens have been widely championed. In 2021, WHO Guidelines on HIV included, for the first time, a Good Practice Statement on patient‐centred services [6].

While the premise of patient‐centredness in the HIV response is increasingly accepted, advancing the use of such practices depends on a collective and coordinated empirical scientific agenda to test the effects of various aspects on patient outcomes. At present, PCC interventions including seemingly distinct practices (e.g. patient empowerment, healthcare worker communications)—what do they mean in total? Furthermore, a particular approach might be more relevant for a particular cascade step (e.g. rapport is more important for retention than testing). In addition, some domains of patient‐centredness have received much more attention than others. DSD and other delivery models have received more attention than those, as put forth by Scholl, related to provider−patient interaction and patient experience [5, 6, 7, 8, 9]. The field also needs to identify the effects of different aspects of patient‐centredness on particular cascade targets (e.g. testing, retention). Prior systematic reviews of PCC have examined alcohol use [7], dementia [8, 9], stroke [10], heart failure [11] and related care quality [12, 13], but the focus on the distinctive needs of people affected by HIV does not yet exist.

To advance the scientific agenda around PCC in HIV, we seek to review and synthesize research on the patient−provider interactions aspects of patient‐centredness in HIV service delivery. We view the diversity of PCC services as distinct enough to make traditional meta‐analysis unhelpful, and instead focus on mapping the types of PCC interventions tested, their feasibility and their outcomes on specific HIV outcomes. Given the fact that many studies have focused on delivery models (e.g. DSD, tuberculosis integration) [3, 14, 15], we focus on domains related to the patient−provider relationship, conceptualized at the core of PCC in multiple models [16], but less examined in HIV care, especially in low‐ and middle‐income countries (LMICs). The provider−patient relationship includes responsiveness to patients’ unique needs, values and preferences; integration of a patient's social, psychological and physical perspectives within a healthcare system; and a collaborative relationship between patients and providers [2, 17, 18]. This review offers a window into the research landscape, areas lacking attention, promising approaches and cascade targets, and will help inform future research investments.

## 2 METHODS

### 2.1 Search strategy

A medical librarian searched the literature guided by PCC domains from the Integrative Model of Patient‐Centeredness by Scholl [16] (Table S1) and included concepts of HIV, LMIC, patient providers, healthcare workers, training programmes, clinician attitudes, interventions, communication and stigmatized patient populations. The librarian created search strategies using a combination of keywords and controlled vocabulary in Embase.com 1947‐, Ovid Medline 1946‐, Scopus 1823‐, Cochrane Central Register of Controlled Trials (CENTRAL), The Cochrane Database of Systematic Reviews (CDSR), Cumulative Index to Nursing and Allied Health Literature (CINAHL) Plus 1937‐ and Clinicaltrials.gov 1997‐. On 1 October 2023, the phrase “person‐centred” was added to all database searches, searches were updated and all new results including newly published citations were added to Covidence [19] where they were de‐duplicated against the existing citations to add 492 new results to the systematic review. We also searched conference abstract archives from IAS‐International AIDS Society (both the International AIDS Conferences and the IAS Conference on HIV Pathogenesis, Treatment and Prevention [IAS] [IAS Conference on HIV Science since 2017]); the International Association of Providers of AIDS Care (IAPAC) International Conference on HIV Treatment and Prevention Adherence (Adherence) and the International Conference on AIDS and STIs in Africa (ICASA) for the following years: IAS/AIDS 2001−2022; IAPAC Adherence: 2010−2021; ICASA 2015, 2017, 2019, 2021. In addition, from January to April 2021, the team conducted iterative, targeted searches on six categories of PCC interventions (see 2.3). The PCC intervention category name and relevant targeted search terms were derived through a review of included articles from a database search conducted in 2019 and study team member dialogue. Targeted searches were conducted in Google and PubMed. Study team members conducted screening and extraction consistent with procedures used for secondary references. Most results were subsequently captured in the January 2023 search. We reviewed the references of all included studies to identify possible additional relevant studies. We deleted duplicate records following standard de‐duplication procedures.

### 2.2 Study eligibility

All study designs were eligible for inclusion. Our study population included persons of any age living with HIV in an LMIC [20]. For the intervention, we included PCC interventions acting at the level of patient−provider interactions seeking to enhance one or more of the following domains of PCC included in the Scholl et al. [16] model: essential characteristics of the clinician, clinician−patient relationship, patient as a unique person, biopsychosocial perspective, clinician−patient communication, patient information, patient involvement in care, involvement of family and friends, patient empowerment, physical support and emotional support (Table S1). The PCC interventions also had to be consistent with the WHO 2015 conceptualization of people‐centred health services [4]. Patient−provider interactions were further operationalised as interactions between a patient and a healthcare provider or primary HIV service provider (excluding lay health workers) based either at the facility or community level. Interventions were excluded if they: (1) sought to solely improve provider clinical expertise, competency or application of evidence‐based practice; (2) addressed solely HIV care “architecture” such as access to care and treatment, and not the patient−provider relationship, thus excluding interventions solely employing differentiated service delivery or care integration; or (3) addressed solely HIV prevention. Studies of multicomponent interventions were included if at least one component enhanced the relevant PCC domains (above), and the study was otherwise consistent with eligibility criteria. We excluded studies that reported uniquely on outcomes from healthcare workers’ perspectives and prevention behaviours such as condom use. The primary outcomes included HIV care continuum outcomes: linkage to HIV treatment services, linkage to antiretroviral therapy (ART), retention in care, adherence to ART and viral suppression. The secondary outcomes included person‐reported outcomes (PROs) defined as the patient's health status originating directly from the patient [21, 22]. We further categorized PROs into the following categories: (1) patient−provider communication; (2) patient satisfaction; (3) perceived quality of care; (4) economic‐related outcomes; and (5) others.

### 2.3 Data collection and synthesis

At least two review authors screened each title and abstract of identified references to determine whether a full‐text review was warranted and proceeded to read the full manuscript to assess eligibility. The review authors discussed disagreements and made final decisions using consensus. At least one review author extracted data from each included article into the LIVE database [23] which uses the Airtable platform [24], and verified the entered information. Abstracted variables included study location, study design, study dates, duration of study and follow‐up, number and type of sites, characteristics of the study population, description of the intervention and comparison group, outcomes, intervention implementation including actors, actions, action targets, dose, location and mechanism; intervention dimensions based on Scholl et al. model [16], and risk of bias assessments. These dimensions were reviewed by two additional review authors who resolved discrepancies through dialogue. After synthesis of our initial results, to better characterize intervention types, we inductively identified five categories of PCC interventions in our results: (1) improved individualized counselling and patient‐centred communication; (2) provider sensitisation to patient experiences and identities; (3) providing friendly and welcoming services; (4) patient empowerment and improved communication skills; and (5) audit and feedback. Interventions were further assigned a main PCC component as agreed by two reviewer authors.

We conducted an independent synthesis of both quantitative and qualitative data. We conducted a qualitative thematic synthesis and presented narrative summaries of qualitative research where appropriate. For quantitative studies, we did not perform a meta‐analysis due to the heterogeneity of the PCC interventions and study methods. For studies with interim and end‐of‐study outcomes, we used the end‐point study outcomes.

### 2.4 Assessment of risk of bias

We used the following risk of bias tools: Cochrane Risk of Bias [25] for randomized trials, the Newcastle‐Ottawa Scale [26] for observational studies, Joanna Briggs Institute critical appraisal checklist for qualitative studies [27] and the Mixed Methods Appraisal Tool (MMAT) for mixed methods studies [28].

## 3 RESULTS

### 3.1 Literature search results

**Figure 1:**
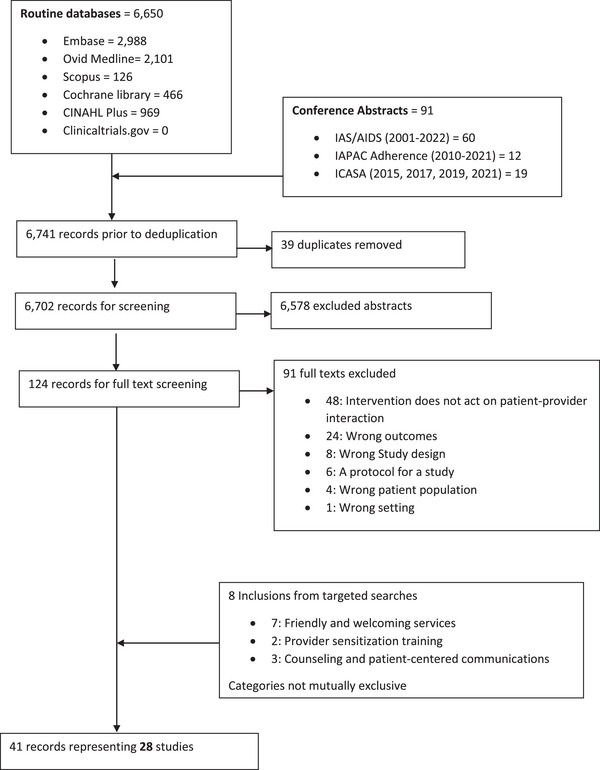
Flow diagram.

The electronic search yielded 6650 records. Ninety‐one additional records were identified from conference abstracts (*n* = 6741) (Figure 1). We removed 39 duplicates and reviewed 6702 titles and abstracts and 124 were eligible for full‐text review. The strategic search yielded eight additional references. From full‐text review, we identified 41 records (manuscripts or abstracts) representing 28 unique studies meeting our inclusion criteria. The SEARCH trial [29] included four unique study reports (different study populations or outcomes) [30, 31, 32, 33]. The Community Score Card study had two unique study reports [34, 35].

### 3.2 Characteristics of studies

Of the 28 studies included, 18 (64.3%) unique studies were quantitative [29, 36, 37, 38, 39, 40, 41, 42, 43, 44, 45, 46, 47, 48, 49, 50, 51, 52], eight were mixed‐methods (28.6%) [35, 53, 54, 55, 56, 57, 58, 59] and two (7.1%) were qualitative studies [60, 61] (Table 1). Overall, 25 studies were conducted in Africa and three from Latin America and the Caribbean [40, 46, 58]. Most studies included adults (46.4%, *n* = 13) [29, 37, 38, 39, 40, 41, 50, 52, 53, 54, 56, 58, 62], followed by adolescents, or adolescents and young adults (28.6%, *n* = 8) [36, 43, 44, 45, 46, 47, 48, 59], pregnant or breastfeeding women (10.7.5%, *n* = 3) [35, 49, 63], sex workers (7.1%, *n* = 2) [42, 57], and children and adults (7.1%, *n* = 2) [29, 61]. In 89% (25/28) of the studies, women represented most of the study population. Among the unique quantitative studies (*n* = 18), seven were pre‐ and post (38.9%) [36, 43, 45, 47, 48, 49, 52], three (16.7%) were observational cohort studies [37, 41, 46], three were cluster‐randomized controlled trials (cRCTs) (16.7%) [42, 44, 64], three were RCTs (16.7%) [39, 40, 50], one was cross‐sectional (5.6%) [38] and one was a controlled before and after study (5.6%) [65]. There were three study reports from the SEARCH trial [29] that used a cohort study design [30, 31, 33]. For the quantitative and mixed methods studies (*n* = 26), the sample size varied from 60 to 6190.

**Table 1:** Characteristics of the included studies

Study		Country	Population	Sample size	Study design	Comparison	PCC intervention description	Patient−provider intervention component category	Scholl dimensions^a^ [16]
SEARCH Trial	Ayieko et al. [31]	Uganda, Kenya	Adults	2051	Prospective cohort	None	Streamlined care intervention in community clinics (PCC, efficient visits, clinic access, reminders and counselling).	Friendly services^b^	HCW−Pt relation; HCW−Pt Comm; Access care; Coord care; Pt info; Phys support
	Balzer et al. [33]	Uganda, Kenya	Children (2−14 y)	665	Prospective single arm	Baseline			
	Jakubowski et al. [30]	Uganda, Kenya	Adults	2796	Prospective cohort	Baseline; control communities			
	Hickey et al. [32] (Brown et al. [88], Havlir et al. [89], Kamya et al. [90], Kwarisiima et al. [51])	Uganda, Kenya	Adults	6190	cRCT	SOC			
Abboah‐Offei et al.^c^ [53]		Ghana	Adults	60	Mixed methods	SOC	HCP received training programme on PCC and communication; participants receive clinical care from the trained HCP.	Imp indiv counselling^b^	HCW−Pt Comm; Biopsych; Pt involve
Emerenini et al. [36]		Nigeria	Adolescents and YA (10−24 y)	4210	Pre‐post	Historical control	Provide adolescent‐friendly services.	Friendly services^b^; HCW sensitisation; Imp indiv counselling	HCW−Pt relation; HCW−Pt Comm; Access care; HCW character; Biopsych; Fam involve; Pt empowerment
Erb et al.^c^ [37]		Tanzania	Adults	299	Prospective cohort	Baseline	Provider education and training on Pt‐centred communication and the provision of an adherence assessment checklist.	Imp indiv counselling^b^	HCW−Pt relation; HCW−Pt Comm; Pt involve
Galy et al. [38]		Cameroon	Adults	146	Cross‐sectional	None	Consultations on knowledge and skills related to HIV management, deciding on educational goals, use of tools to educate, and then evaluation of educational objectives.	Pt empowerment; Imp indiv counselling^b^; Friendly services	Pt empowerment; Pt info; Pt involve; HCW−Pt relation; Biopsych; Pt unique; HCW−Pt Comm; Emo support; Integration
Jones et al.^c^ [39]		Zambia	Adults	160	RCT	Crossover	Pts trained in health literacy and medication adherence, effective communication with providers through training sessions.	Pt empowerment^b^	HCW−Pt relation; HCW−Pt Comm; Pt involve; Pt info
Jones et al.^c^ [40]		Argentina	Adults (identified as lost to care)	120	RCT	SOC	Train HCWs in motivational interviewing (stimulate Pt motivation and promote behaviour change).	Pt empowerment; Imp indiv counselling^b^	HCW−Pt relation; HCW−Pt Comm; Pt involve; Pt info; Pt empowerment
Care CSC	Kays et al. [35] (Chauwa 2019)	Malawi	Pregnant/Postpartum women	1233	Mixed methods	Historical control	Community Score Card created to bring Pts and service providers to collectively share feedback and improve the access and quality of vertical transmission services.	HCW feedback^b^	Pt involve; HCW−Pt Comm
	Laterra et al. [34]	Malawi	Pregnant/Postpartum women	822	Pre‐post	Baseline			
Keene^c^ [41]		South Africa	Adults (identified as lost to care)	196	Cohort	None	Providers trained to welcome disengaged Pts back to care, normalize disengagement, support and empower them.	HCW sensitisation; Friendly services^b^	HCW−Pt relation; HCW character; Biopsych; Pt empowerment
Kerrigan et al. [42]		Tanzania	FSW	387	Prospective community randomized trial	Two matched communities	Providers were trained to administer healthcare to meet the unique needs of the FSW population through sensitivity training.	HCW sensitisation^b^	HCW−Pt relation; Biopsych; HCW character; Integration
Lowther et al. [54]		Kenya	Adults	120	Mixed methods (using qual component for this study)	SOC	HIV clinic nurses received 2 weeks of palliative care training to enhance PCC. An experienced local hospice nurse provided the nurses with weekly supervision and mentoring.	Imp indiv counselling^b^	HCW−Pt relation; HCW−Pt Comm; Biopsych; Emo support; Fam involve
MacLachlan et al. [55] (MacLachlan et al. [62])		Namibia	Adults newly initiated on ART	589	Mixed methods	No training during initial intervention phase	Provide Pts with training on how to actively engage with providers, tools for improved Pt‐provider communication and how to overcome barriers to communication.	Pt empowerment^b^	HCW−Pt Comm; Pt empowerment; Pt involve; HCW−Pt relation; Pt info
Mburu et al.^c^ [43]		Kenya	Adolescents (10−19 y)	3540	Pre‐post	Historical control, SOC	Providers trained on providing adolescent tailored health services including developmental, reproductive, sexual, psychosocial and mental health as well as HIV prevention, care and treatment.	Friendly services^b^; HCW sensitisation; Imp indiv counselling	HCW character; HCW−Pt relation; HCW−Pt Comm; Biopsych
Medina‐Marino et al. [60]		South Africa	Pregnant women	28	Qualitative	NA	Nurses trained to provide support to Pts for disclosing their STI status to sexual partners including training in compassionate care and motivational interviewing skills.	Imp indiv counselling^b^	HCW−Pt relation; HCW−Pt Comm; HCW character
Moucheraud et al. [65]		Uganda, Tanzania	Adults	1112	Controlled before and after	Historical control, SOC	Training for providers on how to effectively provide self‐management counselling.	Imp indiv counselling^b^; Pt empowerment	HCW−Pt relation; Pt unique; Biopsych; HCW−Pt Comm; Pt empowerment; Integration; Pt info; Pt involve
Mutambo et al. [61] (Mutambo et al. [91])		South Africa	Children (2−14 y), Adults	30	Qualitative	NA	A combination of healthcare worker capacity building, the creation of child‐friendly spaces and child‐friendly tools to address medical and social needs of children and adolescents.	Imp indiv counselling; Friendly services^b^; HCW sensitisation	HCW‐Pt Comm; HCW character; Fam involve; Pt involve; HCW−Pt relation
Mwangwa et al. [44]		Uganda, Kenya	Adolescents and YA (15−24 y)	1834	cRCT	Baseline	Pts in intervention clinics received life‐stage specific assessment and counselling at the start of routine visits, choice of flexible clinic access HCPs had a secure mobile platform for inter‐provider consultation.	Friendly services^b^	HCW−Pt relation; HCW−Pt Comm; Access care; Coord care; Pt info; Phys support
Onokala et al. [52]		Nigeria	Adult women with advanced HIV	2267	Pre‐post	Historical control	HCWs trained to implement integrated PCC model. Pts and providers collectively set self‐care plans and therapeutic goals.	Imp indiv counselling^b^	HCW−Pt relation; Teamwork
Pascoe et al. [56]		South Africa	Adults	730	Mixed methods	SOC	Pts supported to practice incorporating ART into their daily lives by addressing common barriers to starting ART and cognitive behavioural techniques.	Imp indiv counselling^b^	HCW−Pt relation; Pt involve; Pt info
PATA [45]		Uganda	Adolescents and YA (10−24 y)	1630	Pre‐post	Historical control	A combination of health provider sensitisation, dedicated adolescent‐friendly service spaces, times and location for improved adolescent and YA care.	HCW sensitisation; Friendly services^b^; HCW feedback	HCW character; HCW−Pt relation; Pt unique; Biopsych; Integration; Access care; Pt info; Coord care; Pt involve
Penfold et al. [57]		South Africa	Sex workers	37	Mixed methods	None	Sensitivity training to prepare providers to work with FSWs	HCW sensitisation^b^; Friendly services	HCW character; HCW−Pt relation; Access care
Puttkammer et al. [58]		Haiti	Adults	874	Mixed methods	Historical control	An iSante EMR Alert for risk of treatment failure prompted HCPs to engage in a 5‐step problem‐solving counselling session with their Pts.	Imp indiv counselling^b^; Pt empowerment	HCW−Pt relation; Pt unique; HCW−Pt Comm; Pt empowerment; Pt involve
Reif et al. [46]		Haiti	Adolescents (10−19 y)	50	Prospective cohort	Baseline (for the non‐HIV outcomes)	A monthly group visit consisting of 30 minutes of peer socialization and 30–45 minutes of peer‐facilitated group counselling led by the FANMI nurse and social worker.	Friendly services^b^	Teamwork; HCW−Pt relation; Pt info; Emo support; Biopsych; Access care
Ruria et al. [47]		Kenya	Adolescents and YA (15−21 y)	559	Pre‐post	Historical control	A school‐based programme that included peer‐navigation and psychosocial support on HIV and SRH; creating a supportive environment to ensure ART adherence; supporting linkage to HCFs.	HCW sensitisation; Friendly services^b^	Pt unique; HCW character; HCW−Pt relation; Biopsych; Access care
Smith^c^ [48]		South Africa	Adolescents	ND	Pre‐post	Before‐after	A roving clinical team provided on‐site training to HCWs on adolescent‐friendly services, developing consultation rooms for adolescents to seek support, same‐day ART initiation and ongoing psychosocial support via 1‐on‐1 adherence counselling.	Imp indiv counselling; Friendly services^b^; HCW sensitisation	HCW−Pt relation; HCW character; Emo support; Biopsych
Teasdale et al.^c^ [49]		Eswatini	Pregnant women	508	Pre‐post	Historical control	Pregnant women attending ANC visits took self‐interview surveys. The survey asked about women's interactions with HCWs. Survey results were shared with HFS at monthly quality improvement sessions.	HCW feedback^b^	HCW−Pt relation; Pt involve
Wachira et al.^c^ [50] (Wachira et al. [66])		Kenya	Adults	328	RCT	SOC, intervention ii	HCWs were trained on principles of communication, provider−Pt relationship dynamics and motivational interviewing as well as EPC packaging that included clinic scheduling and treatment dialogue.	Imp indiv counselling^b^; Pt empowerment	HCW−Pt relation; HCW−Pt Comm; Pt involve; Pt unique; Teamwork; Coord care; Pt empowerment; Access care
Zanoni et al. [59] (Zanoni et al. [67])		South Africa	Adolescents and YA (13−24 y)	241	Mixed methods	SOC	HCWs were trained on principles of communication, provider−Pt relationship dynamics and motivational interviewing.	Friendly services^b^	HCW−Pt relation; Biopsych; Access care; Emo support

Abbreviations: cRCT, cluster randomized controlled trial; FSW, female sex worker; HCW feedback, audit and feedback; HCW sensitisation, provider sensitisation to patient experiences and identities; Imp indiv counselling, improved individualized counselling and patient‐centred communication; ND, not described; providing friendly and welcoming services, friendly services; Pt empowerment, patient empowerment and improved communication skills; Pt outreach, additional patient outreach; RCT, randomized controlled trial; SOC, standard of care; y, years old; YA, young adults.

^a^ While studies were only included if they enhanced one of more of the following Scholl model dimensions: “essential characteristics of the clinician, clinician‐patient relationship, patient as a unique person, biopsychosocial perspective, clinician‐patient communication, patient information, patient involvement in care, involvement of family and friends, patient empowerment, physical support, and emotional support,” here we note *all* Scholl dimensions, including the remaining enablers (i.e. integration of medical and non‐medical care; teamwork and team building, access to care; coordination and continuity of care) if they co‐occurred in an included article.

^b^ Main patient−provider intervention component category.

^c^ Intervention designed primarily to improve patient−provider interaction.

### 3.3 Interventions

All interventions acted on more than one Scholl dimension (Tables S2 and S6). Most commonly, studies addressed clinician−patient relationship (92.9%, *n* = 26), followed by clinician−patient communication (60.7%, *n* = 17), patient involvement in care (50.0%, *n* = 14), biopsychosocial perspective (46.6%, *n* = 13), patient‐tailored information (35.7%, *n* = 10), essential characteristics of the clinician (35.7%, *n* = 10), access to care (32.1%, *n* = 9), patient empowerment (28.6%, *n* = 8), patient as a unique person (21.4%, *n* = 6), emotional support (17.9%, *n* = 5), coordination and continuity of care (14.3%, *n* = 4), integration of medical and non‐medical care (14.3%, *n* = 4), teamwork and teambuilding among staff (10.7%, *n* = 3), and involvement of family and friends (10.7%, *n* = 3), and physical support (7.1%, *n* = 2). Most studies included PCC as part of a multicomponent intervention (60.7%, *n* = 17), while the remaining (39.3%, *n* = 11) had the PCC intervention as the primary intervention (Table 1).

Across the included studies, the actors used to implement PCC‐related actions were most commonly healthcare providers, followed by clinic or programme staff, and then patients (Table S4). Overall, 64.2% (18/28) of studies included repeat intervention exposure/delivery, while one‐third had a single dose at baseline or did not describe the intervention dose. Descriptions of theorized or deductive mechanisms of action varied in detail provided in the publications. Importantly, similar interventions were not depicted as working through the same mechanisms of action across studies. For example, provider training in patient‐centred communication in one study was intended to improve provider ability to elicit patient experiences for improved care [37], while in other studies, it was intended to strengthen provider attitude [40] or mutual exchange of information between providers and patients [39].

### 3.4 Quantitative outcomes

For HIV care continuum outcomes, four studies reported linkage to HIV care [31, 42, 47, 48], three studies reported ART initiation [47, 56, 57], six studies reported ART adherence [37, 39, 40, 42, 52, 58], 12 studies included retention [32, 35, 41, 42, 46, 47, 49–50, 53, 56, 59, 65], one study included viral load coverage [36] and 14 studies reported viral suppression [29, 36, 37, 40–46, 50, 56, 58, 59] (Table S3). Among the included studies with a comparison arm and effect size reported, 62.5% (10/16) reported a significant positive effect of the intervention on at least one HIV care continuum outcome. We identified seven studies reporting at least one PRO [30, 34, 38, 40, 46, 53, 55]. Four studies reported patient−provider communication outcomes [34, 40, 53, 55], three studies reported patient satisfaction [38, 40, 53], one study on perceived quality of care [34], one on economic‐related outcomes [30] and three studies on other PROs (Table 3) [40, 46, 53].

#### 3.4.1 Individualized counselling and patient‐centred communication

##### 3.4.1.1 HIV care continuum outcomes

Eight studies included individualized counselling and patient‐centred communication as the main intervention [30, 31, 47, 49, 54, 57–59] and six studies had a comparison group [37, 40, 56, 58, 65, 66]. Among them, three studies (50%) reported that the intervention had a positive effect on at least one of the HIV care continuum outcomes reported [50, 58, 65] (Table 2).

**Table 2:** PCC components by outcomes for studies with a comparison arm

PCC components	Individualized counselling and PC communication (*n*/*N*, %)	Provider sensitisation training (*n*/*N*, %)	Friendly services (*n*/*N*, %)	Pt empowerment (*n*/*N*, %)	HCW feedback (*n*/*N*, %)
**HIV outcomes** ^a^					
Linkage	NA	Positive effect (1/1, 100.0)	Positive effect (2/2, 100.0)	NA	NA
ART initiation	No difference (0/1, 0.0)	NA	No difference (0/1, 0.0)	NA	NA
Adherence	Mixed findings (1/3, 33.3)	Positive effect (1/1, 100.0)	NA	No difference (0/1, 0)	NA
Retention	Mixed findings (1/4, 25.0)	Positive effect (1/1, 100.0)	Positive effect (3/3, 100)	NA	No difference (0/2, 0.0)
Viral suppression	Mixed findings (1/5, 20.0)	No difference (0/1, 0.0)	Mixed findings (6/6, 100.0)	NA	NA
Studies reporting a positive effect on at least one HIV care continuum outcome by PCC component	3/6 (50.0)	1/1 (100.0)	8/8 (100.0)	0/1 (0.0)	0/2 (0.0)
**PROs** ^b^					
Patient−provider communication	Positive effect (2/2, 100.0)	NA	NA	Positive effect (1/1, 100.0)	Positive effect (1/1, 100.0)
Patient satisfaction	Positive effect (2/2, 100.0)	NA	NA	NA	
Perceived quality of care	NA	NA	NA	NA	Positive effect (3/3,100.0)
Other identified mechanisms	Mixed findings (4/7, 57.1)	NA	Mixed findings (2/3, 66.7)	NA	NA
Economic‐related outcomes	NA	NA	Mixed findings (1/4, 25.0)	NA	NA
Studies reporting at least one positive effect on PROs by PCC component	2/2 (100.0)	NA	2/2 (100.0)	1/1 (100.0)	1/1 (100.0)

Abbreviations: ART, antiretroviral therapy; HCW, healthcare worker; PCC, person‐centred care; PC, patient‐centred; PROS, person‐reported outcomes; Pt, patient.

^a^ For HIV outcomes, the numerator is the number of studies reporting a positive effect of the intervention and the denominator is the number of studies reporting the outcomes of interest.

^b^ For person‐reported outcomes, the numerator is the number of outcomes positively associated with the intervention over the total number of PROs reported.

Individualized counselling and decision support with linkage to community services led to an increase in retention in Tanzania (adjusted odds ratio [aOR] = 3.53, 95% confidence interval [CI]: 2.15−5.77) as compared to standard of care, while there was no effect observed in Uganda (aOR = 1.62, 95% CI: 0.37−7.02). As compared to a historical control, provider‐delivered problem‐solving counselling in Haiti had a positive effect on adherence to ART (adjusted incidence rate ratio [aIRR] = 4.00, 95% CI: 1.91−8.38) but no effect on viral suppression (aIRR = 1.15, 95% CI: 0.92−1.45) [58]. In Kenya, an enhanced patient care intervention that included provider training, continuity of clinician−patient relationship, enhanced treatment communication and convenient clinic scheduling was positively associated with viral suppression (aOR: 2.78, 95% CI: 1.39−5.56), while no effect on retention between study's arms which was at 93% and above across the groups [66].

##### 3.4.1.2 PROs

Among the three studies which included PROs, two included a comparison group. Trained healthcare providers on PCC and communication led to a significant change (*p*<0.001) from pre‐ to post‐intervention on all five PROs reported in Ghana. A study conducted in Argentina found that participants who consulted with a provider who was trained and supervised on patient−provider communication reported a higher level of satisfaction (*p* = 0.01) and better communication with provider (*p* = 0.02), while no effect on the three remaining PROs (self‐efficacy, HIV knowledge and motivation for adherence) compared to participants who received services from providers who did not receive the training (Table 3) [40].

**Table 3:** HIV care continuum and patient‐reported outcomes of included studies, by intervention categories

Study	Outcome (definition)	F/up (months)	Intervention group *n*/*N* (%)	Comparison group *n*/*N* (%)	Effect size (95% CI)	*p* value	Key findings
**I. Individualized counselling and patient‐centred communication**							
**ART initiation**							
Pascoe et al. [56]	ART initiation (initiated on ART within 30 days of eligibility)	12	298/360 (83.0)	303/368 (82.0)	aRD: 6.3% (−0.6 to 13.3)	ND	No significant difference between groups.
**ART adherence**							
Erb et al.^a^ [37]	ART adherence (self‐reported ≥ 1 dose missed in the last 4 weeks)	6−9	16/280 (5.7)	10/299 (3.3)	ND	0.2	No significant difference between groups.
Jones et al.^a^ [40]	ART adherence (0−100 rating of percent adherence in the last 4 weeks)	9	Pt + HCW active: Mean (SD): 82.0 (5.6) *n* = 30	Pt active/HCW inactive: Mean (SD): 63.0 (5.6) *n* = 31	ND	0.47	No significant difference between groups.
Puttkammer et al. [58]	ART adherence (high adherence, “proportion of days covered” ≥ 90%)	36	40/362 (11.0) pre	71/366 (19.4) pre	aIRR:4.00 (1.91−8.38)	<0.001	The intervention had a significant positive effect.
			25/81 (30.9) post	11/65 (16.9) post			
Puttkammer et al. [58]	ART adherence (high adherence, never >7 days late for ART pickup)	36	116/362 (32.0) pre	165/366 (45.0) pre	aIRR: 2.16 (1.42−3.28)	<0.001	The intervention had a significant positive effect.
			42/81 (51.9) post	24/65 (36.9) post			
Onokala et al. [52]	ART adherence (missed ≤ 3 doses/month)	48	1195/2267 (88.0)	907/2267 (40.0)	ND	ND	Descriptive: 88% ART initiation in intervention, 40% in control.
**Retention**							
Abboah‐Offei et al.^a^ [53]	Retention into the study	3	30/30 (100.0)	28/30 (93.3)	ND	ND	Descriptive: Retention was high (>90%) across both groups.
Moucheraud et al. [65]	Appointment attendance in Uganda (attended appointment on scheduled day)	18−24	ND/1650	ND/51	aOR: 1.62 (0.37−7.02)	ND	No significant difference between groups.
Moucheraud et al. [65]	Appointment attendance in Tanzania (attended appointment on scheduled day)	18−24	ND/3416	ND/119	aOR: 3.53 (2.15−5.77)	<0.001	The intervention had a significant positive effect.
Pascoe et al. [56]	Retention in care (not transferred, lost‐to‐follow‐up, failure to attend ART visit or died)	12	240/360 (66.7)	254/367 (69.2)	aRD: −3.6% (−11.1 to 3.9)	ND	No significant difference between groups.
Wachira et al.^a^ [50]	Retention in care (two consecutive visits within 7 days of scheduled clinic appointment date)	6	108/110 (98.2)	101/108 (93.3)	aOR: 2.70 (0.56−20.00) (inverse OR)	ND	No significant difference between groups.
Wachira et al.^a^ [50]	Retention in care (two consecutive visits within 7 days of scheduled clinic visit)	6	105/110 (95.1)	101/108 (93.3)	ND	ND	No significant difference between groups.
**Viral suppression**							
Erb et al.^a^ [37]	Viral suppression (≤1000 copies/ml)	6−9	258/284 (90.8)	274/297 (92.3)	OR: 0.92 (NR)	0.5	No significant difference between groups.
Jones et al.^a^ [40]	Viral suppression (not defined)	9	Pt+HCW active 14/30 (46.7)	Pt active/HCW 15/31 (48)	ND	0.996	No significant difference between groups.
Pascoe et al. [56]	Viral suppression (<400 copies/ml)	12	221/362 (61.1)	235/368 (63.9)	aRD: −1.9% (−9.1 to 5.4)	ND	No significant difference between groups.
Puttkammer et al. [58]	Viral suppression (<1000 copies/ml)	36	37/43 (86.0) pre	125/143 (87.4) pre	aIRR: 1.15 (0.92−1.45)	0.21	No significant difference between groups.
			36/45 (80.0) post	43/56 (76.8) post			
Wachira et al.^a^ [50]	Viral suppression (< 400 copies/ml)	6	93/110 (84.4)	80/108 (64.4)	aOR: 2.78 (1.39−5.56) (inverse OR)	<0.05	The intervention had a significant positive effect.
Wachira et al.^a^ [50]	Viral suppression (<400 copies/ml)	6	92/110 (83.7)	80/108 (64.4)	ND	<0.05	The intervention had a significant positive effect.
**PCC outcome—patient**−**provider communication**							
Abboah‐Offei et al.^a^ [53]	Consultation and relational empathy (CAREM)	3	Mean (SD): 33.0 (1.4) *N* = 28	Mean (SD): 7.0 (1.6) *N* = 30	MD: 1.0 (0.45–1.55)	<0.001	The intervention had a significant positive effect.
Jones et al.^a^ [40]	Provider communication and actions related to the treatment^a^	9	Mean (SD): 11.3 (0.5) *N* = 60	Mean (SD): 9.5 (0.6) *N* = 60	ND	0.02	The intervention had a significant positive effect.
**PCC outcome—patient satisfaction**							
Abboah‐Offei et al.^a^ [40]	Patient experience questionnaire (PEQ) Higher score = better outcomes.	3	Mean (SD): 24.0 (2.4) *N* = 28	Mean (SD): 32.0 (1.5) *N* = 30	*d* ^b^: 0.8 (0.27−1.31)	<0.001	The intervention had a significant positive effect.
Jones et al.^a^ [40]	Satisfaction with provider relationship^c^	9	Mean (SD): 14.2 (0.4) *N* = 60	Mean (SD): 12.6 (0.5) *N* = 60	ND	0.01	The intervention had a significant positive effect.
Galy et al. [38]	Satisfaction with the intervention^c^	ND	ND/139	NA	NA	NA	Descriptive: 88.8% of participants were satisfied with the intervention.
**PCC outcome—other**							
Abboah‐Offei et al.^a^ [53]	Person‐centred measures (PO) Higher scores = better outcomes.	3	Mean (SD): 30.0 (3.9) *N* = 28	Mean (SD): 16.0 (3.8) *N* = 30	*d* ^b^: 0.7 (0.17−1.23)	<0.001	The intervention had a significant positive effect.
Abboah‐Offei et al.^a^ [53]	Health‐related quality of life (MOS‐HIV scale) Higher scores = better outcomes.	3	Mean (SD): 83.0 (2.9) *N* = 28	Mean (SD): 53.2 (3.9) *N* = 30	*d* ^b^: 0.7 (0.17−1.23)	<0.001	The intervention had a significant positive effect.
Abboah‐Offei et al.^a^ [53]	Physical, psychological symptoms, spiritual practical and emotional concerns, and psychosocial needs (APOS). Lower scores = better outcomes	3	Mean (SD): 9 (1.7) *N* = 28	Mean (SD): 14 (1.7) *N* = 30	*d* ^b^:0.7 (0.17−1.23)	<0.001	The intervention had a significant positive effect.
Jones et al.^a^ [40]	Self‐efficacy (HIV treatment adherence self‐efficacy scale).	9	Mean (SD): 105.2 (3.8) *N* = 60	Mean (SD): 99.8 (4.3) *N* = 60	ND	0.31	No significant difference between groups.
Jones et al.^a^ [40]	HIV‐related knowledge (adapted from Personal HIV knowledge measure)	9	Mean (SD): 7.7 (0.2) *N* = 60	Mean (SD): 7.6 (0.2) *N* = 60	ND	0.47	No significant difference between groups.
Jones et al.^a^ [40]	Motivation for adherence (LifeWindows Information Motivation Behavioural Skills ART Adherence Questionnaire).	9	Mean (SD): 35.4 (1.1) *N* = 60	Mean (SD): 36.0 (1.2) *N* = 60	ND	0.26	No significant difference between groups.
Jones et al.^a^ [40]	Depression (Beck Depression Inventory II)	9	Mean (SD): 3.3 (0.6) *N* = 60	Mean (SD): 1.4 (0.6) *N* = 60	ND	0.026	The intervention had a significant positive effect.
**II. Provider sensitisation training**							
**Linkage to HIV care**							
Kerrigan et al. [42]	Linkage to HIV care (ever linked to HIV care)	18	72/91 (79.1)	44/80 (55.0)	RR: 1.44 (CI: ND)	0.002	The intervention had a significant positive effect.
**ART initiation**							
Penfold et al. [57]	ART initiation (ND)	48	509/ND post	69/ ND (pre)	ND	ND	No denominator
**Adherence to ART**							
Kerrigan et al. [42]	ART adherence (within the last 4 days)	18	65/91 (71.4)	37/80 (46.2)	RR: 1.54 (CI: ND)	<0.01	The intervention had a significant positive effect.
**Retention**							
Kerrigan et al. [42]	Retention in care (in care during last 6 months)	18	70/91 (76.9)	41/80 (51.2)	RR 1.5: (CI: ND)	<0.001	The intervention had a significant positive effect.
**Viral suppression**							
Kerrigan e al. [42]	Viral suppression (<400 copies/ml)	18	46/91 (50.6)	36/80 (47.4)	RR 1.05 (ND)	0.74	No significant difference between groups.
**III. Providing friendly and welcoming services**							
**Linkage to HIV care**							
Ayieko et al. [31] (SEARCH)	Linkage to HIV care (first HIV clinic visit after the community‐based test)	12	1503/2051 (73.3)	NA	NA	NA	Descriptive: ¾ of individuals were linked to care (overall).
Ayieko et al. [31] (SEARCH)	Linkage to HIV care (linked to care within 7 days of HIV diagnosis)	7 days	1019/2051 (49.7)	NA	NA	NA	Descriptive: 50% were linked to care within 1 week.
Ruria et al. [47]	Linkage to HIV care (completed first appointment with an HIV care provider following a positive HIV test)	18	544/559 (97.3)	222/393 (56.5)	ND	< 0.0001	The intervention had a significant positive effect.
Smith^a^ [48]	Linkage to HIV care (ND)	8	98% post	63% pre	ND	ND	98% in post‐intervention versus 63% in pre‐intervention
**ART initiation**							
Ruria et al. [47]	ART initiation within 1 month	18	430/544 (79.0)	160/222 (72.1)	ND	*p*>0.05	No significant difference between groups.
**Retention**							
Hickey et al.^d^ [32]	Time in care defined as a proportion of follow‐up time that patients were in care (ART experience with baseline viraemia)	36	27/330 (81.8)	173/238 (72.7)	RR: 1.11 (1.02−1.19)	ND	The intervention had a significant positive effect.
Hickey et al.^d^ [32]	Time in care—as a proportion of follow‐up time that patients were in care (ART naïve with baseline CD4<350)	36	380/514 (74.0)	235/351 (67.0)	RR: 1.10 (1.03−1.17)	ND	The intervention had a significant positive effect.
Hickey et al.^d^ [32]	Time in care defined as a proportion of follow‐up time that patients were in care (ART experienced with baseline VS)	36	1416/1646 (86.0)	1063/1312 (81.0)	RR: 1.07 (1.01−1.13)	ND	The intervention had a significant positive effect.
Keene^a^ [41]	Retention in care (not defined)	ND	171/196 (87.2)	NA	NA	NA	Descriptive: 87% retention
Reif et al. [46]	Retention in care (clinic visit between 11 and 13 months from HIV testing)	12	43/50 (86.0)	NA	NA	NA	Descriptive: 86% retention
Ruria et al. [47]	Retention in care (being in care with a record of having been dispensed ART at the time of the evaluation)	6	144/146 (98.6)	117/215 (54.4)	ND	<0.0001	The intervention had a significant positive effect.
Zanoni et al. [59]	Retention in care (one clinic visit or pharmacy refill)	67	84/88 (95.5)	130/153 (85.0)	OR: 3.7 (1.2−11.1)	0.018	The intervention had a significant positive effect.
**Viral load coverage**							
Emerenini et al. [36]	Viral load coverage (not defined)	6	3340/4294 (77.8) (all age groups)	977/3298 (29.6)	ND	<0.001	The intervention had a significant positive effect.
**Viral suppression**							
Balzer et al. [33] (SEARCH)	Viral suppression (<500 copies/ml)	24	412/665 (62.0)	233/665 (35.0)	ND	ND	Descriptive: HIV viral suppression increased from 35% to 62%.
Emerenini et al. [36]	Viral suppression (not defined)	6	3341/3977 (84.0)	1831/2828 (64.8)	ND	0.03	The intervention had a significant positive effect.
Hickey et al. [32] (SEARCH)	Viral suppression (<500 copies/ml)	36	2373/2644 (90.0)	1970/2264 (87.0)	RR: 1.03 (1.01−1.06)	ND	The intervention had a significant positive effect.
Keene^a^ [41]	Viral suppression (<1000 copies/ml)	3	36/46 (78.3)	NA	NA	NA	Descriptive: 78% viral suppression
Mwangwa et al. [44]	Viral suppression (<400 copies/ml)	24	890/915 (87.9)	734/918 (80.0)	aRR: 1.10 (1.03−1.16)	0.002	The intervention had a significant positive effect.
Mburu et al.^a^ [43]	Viral suppression (<1000 copies/ml)	ND	958/1345 (71.2)	1435/2195 (65.4)	aOR: 0.97 (0.72−1.30)	0.84	No significant difference between groups.
Mburu et al.^a^ [43]	Viral suppression (<1000 copies/ml)	ND	1273/1345 (94.6)	1315/2195 (59.9)	aOR: 1.86 (1.04−3.32)	0.04	The intervention had a significant positive effect.
PATA [45]	Viral suppression (not defined)	12	ND/ND (88.4) post	ND/ND (80.0) pre	ND	ND	Descriptive: An increase in viral suppression from 80% to 88.4%.
Reif et al. [46]	Viral suppression (<1000 copies/ml)	12	13/40 (32.5)	NA	NA	NA	Descriptive: 32.5% viral suppression
Zanoni et al. [59]	Viral suppression (<400 copies/ml)	6	80/88 (90.0)	116/153 (75.8)	OR: 2.5 (1.1−5.8)	0.028	The intervention had a significant positive effect.
**PCC outcome—other**							
Reif et al. [46]	Hopelessness: the proportion of adolescents feeling hopeless in the past 30 days.^c^	12	20% (9/45)	38% (19/50)	ND	0.05	The intervention had a significant positive effect.
Reif et al. [46]	Depression: the proportion of individuals who reported depression^c^	12	17% (8/45)	34% (17/50)	ND	0.07	No significant difference between groups.
Reif et al. [46]	Desire for emotional support: the proportion of adolescents reporting a desire for more emotional support from family and friends^c^	12	50% (22/45)	94% (47/50)	ND	<0.01	The intervention had a significant positive effect.
**PCC economic outcomes**							
Jakubowski et al. (SEARCH) [30]	Probability of sought healthcare for illness or injury in the previous 30 days.	36	ND/1387	ND/1409	MD: −10.3 (−22.0 to 0.1)	ND	Pts in intervention communities were 10.3% less likely to report seeking out healthcare for illness or injury.
Jakubowski et al. (SEARCH) [30]	Probability of lost time from activities due to illness in the previous 30 days.	36	ND/1387	ND/1409	Difference: −7.1% (−17.7 to 0.7)	ND	Pts in intervention communities were 7.1% less likely to report loss of time from activities.
Jakubowski et al. (SEARCH) [30]	Probability of increased employment (any work) in the previous 7 days.	36	ND/1387	ND/1409	MD: 9.7 (2.1−18.3)	ND	Pts in the intervention communities were 9.7% more likely to be employed.
Jakubowski et al. (SEARCH) [30]	Probability of spending any money seeking or receiving healthcare in the past 30 days.	36	ND/1387	746/1409 (18.4)	Difference: −12.7% (−22.4 to 0.6)	ND	Pts in intervention communities were 7.1% less likely to report loss of time from activities.
**IV. Training patient in empowerment and communication skills**							
**Adherence to ART**							
Jones et al.^a^ [39]	ART adherence (self‐reported no missed doses in the previous 3 months)	6	52/77 (67.5) [group intervention]	48/83 (57.8) [individual intervention]	ND	0.91	No significant difference between groups.
**PCC outcome—patient−provider communication**							
MacLachlan et al.^a^ [55]	Patient/provider interactions (Roter Interaction Analysis System [RIAS] code to quantify patient−provider conversations)	6	Mean (SD): 2.10 (1.53) *N* = 160	Mean (SD): 1.05 (1.44) *N* = 129	aRD: 0.48 (0.11−0.85)	0.012	The intervention had a significant positive effect.
**V. Feedback to health workers regarding patient concerns and evaluations of service quality**							
**Retention**							
Kays et al. [35]	Retention in vertical transmission services (attended the most recent scheduled visit within 6 months)	6	431/606 (71.1)	352/627 (56.1)	ND	0.53	No significant difference between groups.
Teasdale et al.^a^ [49]	Retention in care (retained in antenatal care within 1‐month window before and after retention endpoint)	6	42/56 (75.0)	29/41 (70.7)	ND	0.54	No significant difference between groups.
**PCC outcome—patient−provider communication**							
Laterra et al. [34]	Patient−provider communication (disclosure support and maintenance of confidentiality of HIV status).^c^	12	69 points post	42 points pre	64% increase	<0.05	The intervention had a significant positive effect.
**PCC outcome—perceived quality of care**							
Laterra et al. [34]	Quality and professionalism of the provider (attitude and commitment of vertical transmission service providers).^c^	12	85 points post	58 points pre	47% increase	≤0.05	The intervention had a significant positive effect.
Laterra et al. [34]	Quality and professionalism of the provider (prevalence of stigma and discriminatory behaviours towards women living with HIV).^c^	12	80 points post	49 points pre	63% reduction	≤0.05	The intervention had a significant positive effect.
Laterra et al. [34]	Perceived quality of care (convenient and timely access to services).^c^	12	86 points post	64 points pre	34% increase	≤0.05	The intervention had a significant positive effect.

Abbreviations: aDRD, adjusted difference in risk differences; aD, adjusted difference; aOR, adjusted OR; aRD, adjusted risk difference; aRR, adjusted risk ratio; ART, antiretroviral therapy; CI, confidence interval; F/up, follow‐up; HCW, health care worker/provider; HR, hazard ratio; IRR, incidence rate ratio; MD, mean difference; NA, not applicable; ND, not described; *n*, number of events; *N*, number of participants; OR, odds ratio; Pt, patient; RD, risk difference; RR, risk ratio; SD, standard deviation; SE, standard error; VS, versus.

^a^ PCC is the main intervention component.

^b^ Cohen's *d* reported in the manuscript, where *d* = 0.2 is a small effect size, *d* = 0.5 is medium and *d* = 0.8 is large.

^c^ PCC measurement not standardized, or no information provided on the measurement.

^d^ Data provided for each group but not overall.

#### 3.4.2 Provider sensitisation training

##### 3.4.2.1 HIV care continuum outcomes

Two studies included provider sensitisation training [42, 57] as the main intervention including one with a comparison group (Table 2). In a community randomized trial, a multi‐component intervention for female sex workers with repeated sensitisation training for healthcare workers in Tanzania led to an improvement in linkage to care (risk ratio [RR]: 1.44, 95% CI: not described [ND], *p*<0.002), adherence (RR = 1.54, 95% CI: ND, *p*<0.01) and retention (RR = 1.5, 95% CI: ND, *p*<0.001), while no effect on viral suppression (RR = 1.05, 95% CI: ND, *p* = 0.74) compared to the standard of care [42].

#### 3.4.3 Providing friendly and welcoming services

##### 3.4.3.1 HIV care continuum outcomes

Eleven studies provided friendly and welcoming services as the main intervention [29, 36, 41, 43–48, 59, 61] including eight studies with a comparison arm [32, 36, 43–45, 47, 48, 59] that all reported the intervention had a positive effect on at least one of the HIV care continuum outcomes compared to the comparison arm [32, 36, 43–45, 47, 48, 59] (Table 2).

Four studies found a positive association between adolescent youth‐friendly interventions and HIV care continuum outcomes [36, 44, 47, 59]. In Kenya, a multi‐component intervention, which included an adolescent‐friendly intervention, improved linkage to HIV care (97% vs. 57%; *p*<0.0001) and retention (98.6% vs. 54.4%, *p*<0.001) compared to the pre‐intervention period [47]. However, there was no significant effect on ART initiation (79% vs. 72%; *p*>0.05) [47]. Compared to the standard of care, adolescents in the SEARCH Youth intervention in Uganda and Kenya reported higher viral suppression (aRR = 1.10, 95% CI: 1.03−1.16) [44]. Similarly, in Nigeria, an adolescent and youth‐friendly services delivery model led to a higher viral suppression (84% vs. 65%, *p* = 0.03) following the implementation of the intervention compared to the pre‐intervention [36]. In South Africa, Zanoni et al. found that the clinic offering adolescent‐friendly services had higher retention (OR = 3.7, 95% CI: 1.2−11.1) and viral suppression (OR = 2.5, 95% CI: 1.1−5.8) compared to a clinic offering the standard of care (SOC) [67]. In Kenya, Mburu et al. found adolescents attending clinics offering adolescent‐friendly clinic days were significantly more likely to be virally suppressed compared to adolescents receiving HIV care in facilities not offering specialized clinic days (OR: 1.86, 95% CI: 1.04−3.32). However, there was no association between the use of an adolescent‐friendly services package and viral suppression (aOR = 0.97, 95% CI: 0.72−1.30) [43]. For adults, a community‐based intervention with outreach services designed to streamline HIV care (SEARCH trial) significantly improved retention across three different sub‐populations (no overall effect size was included) and viral suppression (RR: 1.03, 95% CI: 1.01−1.06) compared to standard of care [32]. The remaining SEARCH studies were descriptive and showed an improvement in the HIV care continuum reported (no effect size provided). In South Africa, Smith found a higher linkage to care following the implementation of multi‐level intervention centred on delivering adolescent‐friendly services compared to pre‐intervention (98% vs. 63%) [48]. As part of the SEARCH study (Uganda and Kenya), with streamlined, community‐based HIV services, adolescents had a higher viral suppression in year 2 compared to baseline (62% to 35%) [33]. Similarly, work from the Paediatric AIDS Treatment for Africa (PATA) reported an improvement in viral suppression following the implementation of youth‐friendly clinic compared to pre‐implementation (88% vs. 80%) [45].

##### 3.4.3.2 PROs

Participants living in the SEARCH intervention communities were less likely to have (1) lost time from activities due to illness (difference –7.1%, 95% CI: −17.7 to 0.7), (2) sought healthcare for illness or injury (–10.3%, 95% CI: –22.0 to 0.1), (3) spent money on healthcare (–12.7%, 95% CI: –22.4 to 0.6) at 3 years after the intervention although these differences were not statistically significant. Participants were significantly more likely to be employed at 3 years compared to control communities (difference: 9.7%, 95% CI: 2.1−18.3). FANMI (“my family” in Creole), a youth welcoming and friendly services in Haiti for adolescents, led to a significant reduction in feelings of hopelessness (*p*<0.05) and desire for emotional support (*p*<0.01) compared to pre‐intervention. There was no change in self‐reported depression following the intervention compared to pre‐intervention (*p* = 0.07).

#### 3.4.4 Training patients in empowerment and communication skills

##### 3.4.4.1 HIV care continuum outcomes

Jones et al. conducted an RCT in Zambia with a case cross‐over to assess the effects of an individual‐ and group‐based intervention using an Information Motivation Behavioural Skills model to target patient−provider communication and enhance motivation and skills related to ART adherence [39]. They found that 1 month after the cross‐over, adherence to ART was comparable to the baseline (*p* = 0.9).

##### 3.4.4.2 PROs

In Namibia, an intervention focused on training patients with communication skills to interact with providers led to an improvement in patient/provider communication compared to standard of care (aRD: 0.48, 95% CI: 0.11−0.85) [55].

#### 3.4.5 Feedback to health workers regarding patient concerns and evaluations of service quality

##### 3.4.5.1 HIV care continuum outcomes

One unique study which included feedback to health workers regarding patient concerns and evaluations of service quality from pregnant women found no significant difference in retention from pre‐ to post‐intervention (Eswatini: 71%−75%, *p* = 0.54, Malawi: 56%−71%, *p* = 0.53) [34, 35].

##### 3.4.5.2 PROs

The implementation of a Community Score Card in Malawi resulted in a significant improvement in patient−provider communication (*p*<0.05), attitude and commitment of the providers (<0.05), perceived stigma and discrimination of provider towards women living with HIV (*p*<0.05) and perceived quality of care (*p*<0.05) compared to pre‐implementation of the intervention [34].

### 3.5 Qualitative outcomes

Of the 28 studies meeting our criteria, nine studies included qualitative findings, including two studies that had additional publications with qualitative data [62, 67] (Table 4).

**Table 4:** Qualitative HIV care continuum and patient‐reported outcomes of included studies

Study name	Outcome (definition)	Description of outcome findings	Example demonstrative quote for outcome
**ART adherence**			
MacLachlan et al. [62]	Adhere to ARVs following the trainings	From the trainings, patients were more motivated, informed and empowered to adhere.	“I think the whole training was useful because it gave us light and understanding how we should take our medications… It helped me on how I should take my medications and how to take them on time… In Training 1 I enjoyed much on how to take my medications, which means I enjoyed most because I now know how to take my medications and how to speak to my doctor.”—(P3, 31 year‐old female)
**Retention in care**			
**PCC outcome**—**patient−provider communication**			
Abboah‐Offei et al. [54]	Communication among people living with HIV and HCP.	Patients felt more comfortable to discuss their care and concerns with providers in the intervention group and the intervention fostered open communication	“Now I feel much better because I don't have to keep quiet about my symptoms of pain or any other physical problems because if I don't talk about them staff will keep on asking me about my physical health. So now staff always ask me about pain and other problems which I always discuss them with staff and together we decide what will help me better.” Person living with HIV 9
Lowther et al. [54]	Having time to talk during appointments	Participants had time dedicated to talking through their problems and concerns, and help articulating their needs.	“I am better because I was listened to; I was helped. I was given advice, and so I left with something.” ID 108, female, 37 years “Here, we have more time with [the nurses]; they will not see you in a hurry like the other place [standard care clinic], because there are other people waiting. Here you will be seen; you will explain your problem.” ID 108, female, 37 years
Medina‐Marino et al. [60]	Communications and trusting relationships with nurses trained in PCC.	Supported women felt less guilt and more confidence when approaching their treatment plan.	“She spoke to me like. what can I say, like a friend. We were like speaking as [if we were] having a friendly conversation … A person speaking to you friendly, openly. You asking questions, or [them] asking you do you have any questions. It makes … me feel relieved …. Because if a person can give me the space to express what I feel or express what I do not understand about whatever situation I'm in like now. But, she gave me everything I would like [health information needed, and then] okay fine, I understand. Mm.”—25‐year‐old female
Zanoni et al. [67]	Patient−provider communication and relationships	Adolescents reported developing stronger relationships with providers by spending more time with them in after hour services	“[At the paediatric clinic] there was no one who had time to ask you what was going on in your life. They only asked you about your health, not how your life as a whole was going. [The adolescent clinic] helped me a lot because I could talk about anything with [names doctor and counsellors].”—21‐year‐old male currently attending the adolescent clinic.
**PCC outcome—patient satisfaction**			
Kays et al. [35]	Increased satisfaction in services after implementation of the CSC intervention	Results from the FGDs showed many clients reporting increased satisfaction due to a strengthened relationship among HCWs and pregnant/postpartum clients.	“It is true that there is now a great improvement on our relationship, because the meetings we had with CARE tried to establish causes of all misunderstandings we had previously, and how to address those misunderstandings.” (pregnant/postpartum client, post‐intervention).
Pascoe et al. [35]	Feeling supported and more involved in decisions	Patients reported feeling more involved in decisions affecting their care and felt supported by additional counselling.	No quote given
Puttkammer et al. [58]	Acceptability and satisfaction of the intervention	Participants expressed acceptability of the intervention and that the colour‐coding helped with their care.	“Yes, the doctor sent me to the Miss who told me that I have the yellow colour and (she) told me that it wasn't good for me and that I have to take my medications.”
**PCC outcome**—**perceived quality of care**			
Kays et al. [35]	Improvement in clinic service provision	Pregnant/postpartum clients were more aware of services provided and the challenges HCWs faced	“Previously, we had a lot of concerns which lacked a platform where they would be addressed the same with health care workers. However, with the introduction of CSC we had that platform to express ourselves. During these meetings we were able to understand other challenges that exist in the system.”—Pregnant/postpartum client
**PCC outcome**—**other identified mechanisms**			
Mutambo et al. [61]	Transformation of the PHC settings into a therapeutic environment for children	Children reported that the child‐friendly spaces transformed the PHC setting into a welcoming environment, something that was previously lacking.	“We do children's activities. The spaces are very colourful, and they remind us of our school environment, which is very nice because it makes us forget that we are here for HIV. This reduces our fear and discomfort,” 9‐year‐old girl, eThekwini District.
Abboah‐Offei et al. [53]	People living with HIV being partners in care delivery	Care focused on patients as individuals, and they were involved in the planning of their own care and making decisions with the providers.	“The way staff assessed my problems step by step wanting to know all about me and my life outside HIV and more importantly my involvement in care and staff planning my care with me was the main thing that helped me most.” PLWH 7
Mutambo et al. [61]	Child participation and involvement in their own healthcare journey	Child‐friendly spaces were effective for reducing child apprehension towards HCWs providing care.	“I now enjoy going to the health facility because we learn a lot about germs and how to put them to sleep using goodnight medicine. I like my nurse because she makes me laugh and we play games, and sometimes she tells me a story. I also ask her questions when I don't understand and she always asks me how I am feeling,” 5‐year‐old boy, eThekwini District.
MacLachlan et al. [62]	Overcoming psychosocial barriers	Prior to trainings, patients were afraid to speak freely, but patients gained courage to be more engaged with providers.	“According to me, these trainings helped me in communicating with the doctor because at first I was afraid to speak to the doctor but after these trainings it helped me how to express myself when I am with the doctor, they really prepared me.”—(P3, 31‐year‐old female)
MacLachlan et al. [62]	Patient knowledge	The trainings increased their knowledge of HIV‐related topics, which enhanced engagement with providers due to being able to ask specific questions about their health.	“I feel that the trainings have prepared me how to better communicate with the providers because now I can even ask the doctors and nurses about the level of the virus in my body and CD4 counts and they can tell me this information.”—(P9, 30‐year‐old male)
MacLachlan et al. [62]	Active patient self‐efficacy referring to a patient's confidence to engage providers	All patients enhanced their active patient self‐efficacy following the training sessions.	“Yes, there are no challenges [that remain for me in terms of being an active patient]. I still urge those who will attend these trainings that they will not have a problem after the training.. . With me, I think there is nothing [else that I want to ask or say to the doctor but cannot]. When I will have a problem I will ask the doctor, that is the good part of these trainings.”—(P4, 30‐year‐old male)

Abbreviations: ARV, antiretroviral; CARE, Cooperative for Assistance and Relief; FGD, focus group discussion; HCWs, health care workers; PHC, primary health care.

#### 3.5.1 HIV care continuum outcomes

People living with HIV expressed being more confident and motivated to adhere to ART after completing the patient education and empowerment training [55].

#### 3.5.2 PROs

All four studies including outcomes related to increased patient−provider communication reported benefits of the intervention. Patients felt more comfortable discussing their care and concerns with providers following the PCC intervention (healthcare worker training and mentorship on patient−provider communication) [53]. Lowther et al. reported that patients felt listened to, were given advice and nurses had the time to discuss their care after a palliative care intervention [54]. Pregnant women felt supported and had more confidence to ask questions and express their feelings with improved individualized counselling [60]. Adolescents from a youth‐friendly clinic reported a deeper connection and ability to communicate openly with staff compared to adolescents attending the standard clinic [35].

Qualitative PCC outcomes related to patient satisfaction were found in three studies. Kays et al. conducted focus group discussions where many clients felt the intervention allowed discussions on misunderstandings and ultimately improved their care [35]. Patients reported feeling more involved in their care decisions in Pascoe et al. leading to improved patient satisfaction [56]. Puttkammer et al. examined the acceptability of their counselling and patient empowerment intervention with patients expressing that the intervention was helpful for their care [58] (Table 4). Kays et al. included a pre‐post PCC outcome related to perceived quality of care. Participants reported that the community scorecard assisted with understanding challenges in the healthcare system [35].

Three studies reported qualitative outcomes that were categorized as other identified PCC mechanisms. Child‐friendly spaces in Mutambo et al. contributed to reducing fears of using ART and discomfort [61]. Similarly, Abboah‐Offei et al. reported that patients felt like partners in their own care and seen as individuals who could make decisions with the providers [53]. Additionally, MacLachlan et al. found that patients gained the courage to be more engaged with providers by overcoming psychosocial barriers [62].

### 3.6 Quality of the included studies

Of the included studies, most of the RCTs and cRCTs had a high or unclear risk of bias (*n* = 5/6, 83.3%) (Table S5a). For cohort studies, most studies (*n* = 4/7, 57.1%) had a score of five or below over a total of nine (nine being the highest score a study can achieve indicating the best quality or low risk of bias). The Newcastle‐Ottawa Scale is an eight‐question checklist where stars are given when high‐quality standards are identified (Table S5b). For pre‐ and post‐studies, seven out of eight studies (87.5%) had a score of five or below out of nine indicating a high risk of bias. The Newcastle‐Ottawa Scale checklist was also used for the pre‐post studies (Table S5b). Similarly, the one cross‐sectional study had a score of four over a total of nine when the Newcastle‐Ottawa Scale was used (Table S5c). For the qualitative studies, the two studies were included, but both were judged with unclear risk of bias (Table S5d). The mixed‐methods studies were judged as poor or fair risk of bias, with most 6/8 (75.0%) having an overall score of fair. These scores of “poor” or “fair” were quantified based on the MMAT, where five questions are asked to assess the quality for each study design used and an additional five questions looking at the overall quality of the mixed methods design (a total of 15 questions per study). Studies designated as poor had a high risk of bias and those designated as fair had some risk of bias (Table S5e).

## 4 DISCUSSION

This review expands on prior work focusing on the importance of patient−provider interactions in the provision of HIV care by including evidence from LMICs and including both HIV care continuum outcomes and PROs. We identified 28 unique studies from Africa, Latin America and the Caribbean. These studies were diverse regarding PCC intervention types; with five main types identified through an inductive process. Among the included studies with a comparison arm and effect size available, 62.5% (10/16) reported a positive effect of the intervention on at least one reported HIV care continuum outcome. Among the 10 studies with a positive result, six of the study interventions provided friendly services, three focused on improved counselling and one on provider sensitisation. All of the 10 studies with a positive result sought to enhance the clinician−patient relationship PCC domain, with 70% intending to address clinician−patient communication, 60% both taking a biopsychosocial perspective and enhanced care access and 40% focusing on patient empowerment, essential characteristics of the clinician, and the patient as a unique person.

The evidence suggests that interventions focused on providing friendly and welcoming services and improving individualized counselling have a strong potential to improve both HIV‐related and PROs. Interventions with patient empowerment (*n* = 1) or audit and feedback (*n* = 2) to providers as the primary focus are promising, as they improved PROs, but warrant further study, as they did not show improvement in HIV‐related outcomes. Encouraging new results from a pilot study in the Democratic Republic of Congo suggest that integrating an electronic client feedback tool into continuous quality improvement (CQI) processes led to positive outcomes such as reduced wait time and stigma and improved viral load services [68].

More than two‐thirds of studies focused on improving the PCC domain of access to care, coordination and continuity of care, physical support, patient empowerment or the patient as a unique person demonstrated a significant improvement in at least one quantitative outcome. Of the three studies seeking to enhance the involvement of family and friends, all showed a positive improvement in outcome (one quantitative, two qualitative). Other PCC domains upon which studies attempted to act had more limited data or more mixed results.

Most outcomes from the included studies were HIV care continuum outcomes with heterogeneity in outcome definitions, and 25% (*n* = 7) of the studies included PROs [30, 34, 38, 40, 46, 53, 55]. We found a broad range of PROs, all reporting unique PCC measures. HIV programmes in LMICs have predominantly used clinical and biomedical measures such as ART initiation, retention and viral suppression for evaluation. UNAIDS has set the 95‐95‐95 global targets which include 95% of all people who are living with HIV knowing their status, 95% of people who know their status being on treatment and 95% of people on treatment achieving viral suppression by 2025 [69]. This global framework has been instrumental in monitoring progress and facilitating comparisons across countries. However, there is also a growing call to move beyond viral suppression [70, 71] and incorporate patient‐reported measures to provide a more comprehensive view of the person [21]. Incorporating PROs is especially relevant as several countries are nearing or have already achieved the UNAIDS targets [72]. Under these circumstances, detecting a meaningful difference in HIV‐related outcomes, such as viral suppression, would be challenging, as most individuals will have high retention and viral suppression. For instance, in this systematic review, one study [53] found no difference between the study arms on retention which may be explained by the overall high retention (≥93%) at baseline. Incorporating PROs may also provide valuable information on proximal outcomes to complement distal outcomes such as viral suppression. Standardizing a subset of PROs could be also prioritized to facilitate comparison across studies.

Key populations, including gay men and other men who have sex with men, sex workers, transgender people, people who inject drugs and prisoners and other incarcerated people [73], face disproportionate barriers to access quality HIV care, such as stigma and discrimination [74, 75]. Only two studies in our review focused on a key population [42, 57]. One of the included studies showed encouraging results on linkage to HIV care and retention among female sex workers [42]. Another population under‐represented in the review is older people living with HIV [76]. This group represents a substantial proportion of people living with HIV and is expected to grow in subsequent years [77]. For instance, in PEPFAR‐supported countries, 21% of persons living with HIV were 50 years and older [76]. Older adults living with HIV are likely to experience a broad array of health conditions such as non‐communicable diseases requiring integrated health services beyond the clinical management of HIV and HIV‐related diseases [77, 78]. Person‐centred service delivery models for this population have been recognized as urgently needed [79]. Future research is warranted to identify and test PCC interventions on HIV‐related outcomes and PROs for different populations who may experience additional barriers to accessing or receiving health services tailored to their unique needs.

The level of description of the intervention components varied across studies. Multiple studies included a limited description of the intervention. While we categorized interventions under a “main” component, most addressed multiple components (Table S7). Details of interventions are critical to promote transparency, adoption and replicability in other settings. Gaps persist in this area, as highlighted by Hickey et al., who conducted a systematic review on the specification of interventions to address the cascade of HIV care in resource‐limited settings and found that reporting of the different intervention components varied between 42% and 69% [64]. The broader use of tools such as the Template for Intervention Description and Replication (TIDieR) [80] and Standards for Reporting Implementation Studies [81] could enhance the systematic reporting of the characteristics of the interventions. Linking study or programmatic materials to the publication may also assist in providing further information about the intervention. Limited information was also provided on the mechanism of change, or how the intervention is expected to work, for both HIV care cascade outcomes and PROs. Several researchers have argued for the need to specify the processes and mechanisms through which implementation strategies are expected to work which may vary depending on the contextual factors (population, settings) [82, 83].

Our review identified additional PCC intervention efforts that did not meet our review criteria due to outcomes reported or search dates but may be informative for advancing PCC in HIV. These include studies that did not include patient outcomes but assessed the implementation of PCC‐interventions, which are critical to optimize PCC intervention delivery [84, 85]. Other studies included efforts to improve PCC through provider education [85], a common implementation strategy, and interventions acting at the health systems level to alleviate barriers that impact the patient−provider relationship [86]. We also identified studies seeking to advance PCC in HIV prevention [87]. Updated and additional reviews as well as an understanding of ongoing facility and community‐level practices to understand different aspects of PCC interventions are warranted to inform the movement of the HIV field and integrated HIV‐primary care to act on PCC principles.

A key limitation of our approach is that by searching for and including studies based on PCC‐related terminology and focused on patient−provider interactions, we may miss relevant studies that did not describe themselves as being focused on PCC. An alternative approach would have been to sample studies based on the reporting of PROs. However, our chosen approach was preferable since PCC‐related outcome measurements are highly heterogeneous and less common than measurements of HIV care cascade outcomes. We restricted our search to specific conferences and our search likely missed studies published in regional meetings such as the Asia‐Pacific AIDS and Co‐infection Conference. This systematic review focused on primary HIV clinicians who were largely facility‐based. Therefore, we did not include lay workers or community workers. Future research should include PCC among other service providers, such as community lay healthcare workers, counsellors or peer navigators, who play a key role in providing HIV services.

## 5 CONCLUSIONS

It is widely agreed that person‐centredness in the interactions between patients and their health providers is an important principle of quality care. Among published HIV PCC interventions from LMICs, there is heterogeneity in the components of PCC addressed, the actors involved and the expected outcomes. While results are also heterogeneous across both PROs and clinical outcomes, they are largely positive. To translate the principles of PCC consistently and effectively to programmatic practice and supportive policies, a deeper theoretical understanding of the mechanisms of intervention actions is required. Improved consistency of outcome measurement is also needed, especially in PROs, to better understand the effects of PCC interventions in HIV across settings. Theory‐driven interventions specifying intended mechanisms of action and consistent outcome measures will help ensure that interventions cover the optimal range of PCC components based on the realities of a specific context.

## COMPETING INTERESTS

The authors do not have a conflict of interest to report.

## AUTHORS’ CONTRIBUTIONS

LKB, IE‐W and EHG designed the study. LY conducted the search strategy. JL, AA, CK, DPM, and BAAK supported the search strategy and data extraction. LKB, AU, NLT, CGK, GK and MCL independently assessed records for eligibility and extracted the data. LKB, AU, NLT, CGK and MCL wrote the initial draft of the manuscript. All authors critically reviewed and revised the drafts and approved the final version for publication.

## FUNDING

This work was supported by the Bill and Melinda Gates Foundation (INV‐009840 to EHG), the National Institute of Health (K24 AI134413 to EHG), (K01MH130244 to LKB) and IAS. The funders had no role in the study design, data collection and analysis, and final manuscript. This review was partially funded by IAS—the International AIDS Society. It was made possible with financial support from Gilead Sciences, as part of their sponsorship of the IAS Person‐Centred Care programme. IAS has full control over all activities and decisions relating to, and forming part of, the IAS Person‐Centred Care programme.

## DISCLAIMER

The content of this review is solely the responsibility of the authors and does not necessarily represent the official views of the International AIDS Society or Gilead Sciences. Gilead Sciences has not had any input into the content of the review.

## Supporting information


**Table S1**: Literature search strategy
**Table S2**: Dimensions of the Integrative Model of Patient‐Centeredness by Scholl et al. (2014)
**Table S3**: HIV care continuum and PCC outcomes of included studies by outcomes
**Table S4**: Patient‐Centered Care Intervention Components aimed at Patient‐Provider Relationship
**Table S5a**: Risk of bias for included studies – RCT or cRCT
**Table S5b**: Risk of bias for included studies – cohort studies and pre‐post studies
**Table S5c**: Risk of bias for included studies – cross‐sectional studies
**Table S5d**: Risk of bias for included studies – qualitative studies
**Table S5e**: Risk of bias for included studies – mixed methods studies
**Table S6**: Scholl domains by outcomes with the percent of study reports having a positive effect among study reports with a comparison arm
**Table S7**: PCC intervention components; number of studies that have components

## Data Availability

Data derived from public domain resources.
