# Supplementary material for: Person‐centred interventions to improve patient−provider relationships for HIV services in low‐ and middle‐income countries: a systematic review
Source: J Int AIDS Soc. 2024 May 13;27(5):e26258. doi: 10.1002/jia2.26258 (PMC11090778; doi:10.1002/jia2.26258)
Supplement: Supplementary file 1 — Table S1: Literature search strategy Table S2: Dimensions of the Integrative Model of Patient‐Centeredness by Scholl et al. (2014) Table S3: HIV care continuum and PCC outcomes of included studies by outcomes Table S4: Patient‐Centered Care Intervention Components aimed at Patient‐Provider Relationship Table S5a: Risk of bias for included studies – RCT or cRCT Table S5b: Risk of bias for included studies – cohort studies and pre‐post studies Table S5c: Risk of bias for included studies – cross‐sectional studies Table S5d: Risk of bias for included studies – qualitative studies Table S5e: Risk of bias for included studies – mixed methods studies Table S6: Scholl domains by outcomes with the percent of study reports having a positive effect among study reports with a comparison arm Table S7: PCC intervention components; number of studies that have components [file JIA2-27-e26258-s001.docx]

Table of Contents

[S1: Table: Literature search strategy 2](#_Toc160195557)

[S2. Dimensions of the Integrative Model of Patient-Centeredness by Scholl et al. (2014) 14](#_Toc160195558)

[S3. Table: HIV care continuum and PCC outcomes of included studies by outcomes 16](#_Toc160195559)

[S4 Table: Patient-Centered Care Intervention Components aimed at Patient-Provider Relationship 22](#_Toc160195560)

[S5a. Table: Risk of bias for included studies – RCT or cRCT 27](#_Toc160195561)

[S5b. Table: Risk of bias for included studies – cohort studies and pre-post studies 28](#_Toc160195562)

[S5c. Table: Risk of bias for included studies – cross-sectional studies 30](#_Toc160195563)

[S5d. Table: Risk of bias for included studies – qualitative studies 31](#_Toc160195564)

[S5e. Table: Risk of bias for included studies – mixed methods studies 32](#_Toc160195565)

[S6. Table: Scholl domains by outcomes with the percent of study reports having a positive effect among study reports with a comparison arm 33](#_Toc160195566)

[S7. Table: PCC intervention components; number of studies that have components 34](#_Toc160195567)

##

## Table S1: Literature search strategy

| **Date** | **Database** | **Search terms** |
| --- | --- | --- |
| 10.1.23 | Embase | ('Human immunodeficiency virus'/exp OR 'Human immunodeficiency virus 1'/exp OR 'Human immunodeficiency virus 2'/exp OR 'Human immunodeficiency virus infected patient'/exp OR 'Human immunodeficiency virus infection'/exp OR 'antiretroviral therapy'/exp OR 'highly active antiretroviral therapy'/exp OR (‘Human immunodeficiency virus’ OR HIV OR ‘HIV-related’ OR ‘HIV-positive’ OR HIV+):ti,ab,kw OR ((‘aids associated’ OR ‘aids related’) NEAR/3 (lentivirus OR retrovirus OR virus)):ti,ab,kw OR ((‘human immuno deficiency’ OR ‘immunodeficiency associated’ OR ‘lymphadenopathy associated’) NEAR/3 (virus OR retrovirus)):ti,ab,kw OR ((antiretroviral OR ‘anti-retroviral’ OR ‘Anti-HIV’) NEAR/3 (therapy OR agent*)):ti,ab,kw)  AND  ('Afghanistan'/exp OR 'Afghan'/exp OR 'Albania'/exp OR 'Albanian (people)'/exp OR 'Algeria'/exp OR 'Algerian'/exp OR 'American Samoa'/exp OR 'American Samoan'/exp OR 'Angola'/exp OR 'Angolan'/exp OR 'Argentina'/exp OR 'Argentinian'/exp OR 'Armenia'/exp OR 'Armenian (people)'/exp OR 'Azerbaijan'/exp OR 'Azerbaijani'/exp OR 'Bangladesh'/exp OR 'Belarus'/exp OR 'Belarusian (citizen)'/exp OR 'Belize'/exp OR 'Belizean'/exp OR 'Benin'/exp OR 'Beninese'/exp OR 'Bhutan'/exp OR 'Bhutanese'/exp OR 'Bolivia'/exp OR 'Bolivian'/exp OR 'Bosnia and Herzegovina'/exp OR 'Bosnian (citizen)'/exp OR 'Bosniak (people)'/exp OR 'Botswana'/exp OR 'Brazil'/exp OR 'Brazilian'/exp OR 'Bulgaria'/exp OR 'Bulgarian (citizen)'/exp OR 'Burkina Faso'/exp OR 'Burundi'/exp OR 'Cape Verde'/exp OR 'Cambodia'/exp OR 'Cambodian'/exp OR 'Cameroon'/exp OR 'Cameroonian'/exp OR 'Central African Republic'/exp OR 'Central African'/exp OR 'Chad'/exp OR 'Chadian (citizen)'/exp OR 'China'/exp OR 'Chinese'/exp OR 'Colombia'/exp OR 'Colombian'/exp OR 'Comoros'/exp OR 'Congo'/exp OR 'Congolese (Brazzaville)'/exp OR 'Costa Rica'/exp OR 'Costa Rican'/exp OR 'Cote d`Ivoire'/exp OR 'Ivorian (citizen)'/exp OR 'Cuba'/exp OR 'Cuban'/exp OR 'Djibouti'/exp OR 'Dominica'/exp OR 'Dominican (Dominica)'/exp OR 'Egypt'/exp OR 'Egyptian'/exp OR 'El Salvador'/exp OR 'Salvadoran'/exp OR 'Eritrea'/exp OR 'Eritrean'/exp OR 'Eswatini'/exp OR 'Swazi (citizen)'/exp OR 'Ethiopia'/exp OR 'Ethiopian'/exp OR 'Gabon'/exp OR 'Gabonese'/exp OR 'Gambia'/exp OR 'Gambian'/exp OR 'Georgia (republic)'/exp OR 'Georgian (people)'/exp OR 'Ghana'/exp OR 'Ghanaian'/exp OR 'Grenada'/exp OR 'Guatemala'/exp OR 'Guatemalan'/exp OR 'Guinean'/exp OR 'Guinea-Bissau'/exp OR 'Guyana'/exp OR 'Guyanese'/exp OR 'Haiti'/exp OR 'Haitian'/exp OR 'Honduras'/exp OR 'Honduran'/exp OR 'India'/exp OR 'Indonesia'/exp OR 'Indonesian'/exp OR 'Iran'/exp OR 'Iranian people'/exp OR 'Iraq'/exp OR 'Iraqi'/exp OR 'Jamaica'/exp OR 'Jamaican'/exp OR 'Jordan'/exp OR 'Jordanian'/exp OR 'Kazakhstan'/exp OR 'Kazakhstani'/exp OR 'Kenya'/exp OR 'Kenyan'/exp OR 'Kiribati'/exp OR 'Korea'/exp OR 'South Korea'/exp OR 'North Korea'/exp OR 'Korean (people)'/exp OR 'Kosovo'/exp OR 'Kosovar'/exp OR 'Kyrgyzstan'/exp OR 'Kyrgyz (people)'/exp OR 'Laos'/exp OR 'Laotian'/exp OR 'Lebanon'/exp OR 'Lebanese'/exp OR 'Lesotho'/exp OR 'Sotho (people)'/exp OR 'Libyan Arab Jamahiriya'/exp OR 'Libyan'/exp OR 'Madagascar'/exp OR 'Malagasy (people)'/exp OR 'Malagasy (citizen)'/exp OR 'Malawi'/exp OR 'Malawian'/exp OR 'Malaysia'/exp OR 'Malaysian'/exp OR 'Maldives'/exp OR 'Mali'/exp OR 'Malian'/exp OR 'Marshall Islands'/exp OR 'Mauritania'/exp OR 'Mauritanian'/exp OR 'Micronesian'/exp OR 'Mongolia'/exp OR 'Mongolian (people)'/exp OR 'Morocco'/exp OR 'Moroccan'/exp OR 'Mozambique'/exp OR 'Mozambican'/exp OR 'Myanmar'/exp OR 'Burmese'/exp OR 'Namibia'/exp OR 'Namibian'/exp OR 'Nepal'/exp OR 'Nepalese'/exp OR 'Nicaragua'/exp OR 'Nicaraguan'/exp OR 'Niger'/exp OR 'Nigeria'/exp OR 'Nigerian'/exp OR 'Republic of North Macedonia'/exp OR 'Macedonian (citizen)'/exp OR 'Macedonian (people)'/exp OR 'Pakistan'/exp OR 'Pakistani'/exp OR 'Panama'/exp OR 'Panamanian'/exp OR 'Papua New Guinea'/exp OR 'Papua New Guinean'/exp OR 'Paraguay'/exp OR 'Paraguayan'/exp OR 'Peru'/exp OR 'Peruvian'/exp OR 'Philippines'/exp OR 'Filipino (citizen)'/exp OR 'Filipino (people)'/exp OR 'Romania'/exp OR 'Romanian (citizen)'/exp OR 'Russian Federation'/exp OR 'Rwanda'/exp OR 'Rwandan'/exp OR 'Samoa'/exp OR 'Samoan (people)'/exp OR 'Sao Tome and Principe'/exp OR 'Senegal'/exp OR 'Senegalese'/exp OR 'Serbia'/exp OR 'Serbian (citizen)'/exp OR 'Sierra Leone'/exp OR 'Sierra Leonean'/exp OR 'Solomon Islands'/exp OR 'Somalia'/exp OR 'Somali (citizen)'/exp OR 'South Africa'/exp OR 'South African'/exp OR 'South Sudan'/exp OR 'Sri Lanka'/exp OR 'Sri Lankan'/exp OR 'Saint Lucia'/exp OR 'Saint Vincent and the Grenadines'/exp OR 'Suriname'/exp OR 'Surinamese'/exp OR 'Syrian Arab Republic'/exp OR 'Syrian'/exp OR 'Tajikistan'/exp OR 'Tajik (citizen)'/exp OR 'Tanzania'/exp OR 'Tanzanian'/exp OR 'Thailand'/exp OR 'Thai (people)'/exp OR 'Timor-Leste'/exp OR 'Togo'/exp OR 'Togolese'/exp OR 'Tonga'/exp OR 'Tongan'/exp OR 'Tonga (people)'/exp OR 'Tunisia'/exp OR 'Tunisian'/exp OR 'Turkey (republic)'/exp OR 'Turkic people'/exp OR 'Uganda'/exp OR 'Ugandan'/exp OR 'Ukraine'/exp OR 'Ukrainian (citizen)'/exp OR 'Uzbekistan'/exp OR 'Uzbek (citizen)'/exp OR 'Vanuatu'/exp OR 'Viet Nam'/exp OR 'Vietnamese'/exp OR 'gaza strip palestine'/exp OR 'gaza strip'/exp OR 'Yemen'/exp OR 'Yemeni'/exp OR 'Zambia'/exp OR 'Zambian'/exp OR 'Zimbabwe'/exp OR 'Zimbabwean'/exp OR 'Dominican Republic'/exp OR 'Dominican (Dominica)'/exp OR 'Dominican Republic'/exp OR 'Mauritius'/exp OR 'Mauritian'/exp OR 'Turkmenistan'/exp OR 'Equatorial Guinea'/exp OR 'Mexico'/exp OR 'Mexican'/exp OR 'Tuvalu'/exp OR 'Ecuador'/exp OR 'Ecuadorean'/exp OR 'Moldova'/exp OR 'Fiji'/exp OR 'Fijian'/exp OR 'Montenegro (republic)'/exp OR (Afghanistan OR Afghan OR Afghans OR Albania* OR Algeria* OR ‘American Samoa*’ OR Angola* OR Argentina OR Argentinian* OR Armenia* OR Azerbaijan* OR Bangladesh* OR Belarus* OR Belize* OR Benin* OR Bhutan* OR Bolivia* OR ‘Bosnia and Herzegovina’ OR Bosnian* OR Bosniak* OR Botswana OR Bechuanaland OR Brazil OR Brazilian* OR Bulgaria* OR ‘Burkina Faso’ OR ‘upper volta’ OR Burundi OR ‘Cabo Verde’ OR ‘Cape Verde’ OR Cambodia OR kampudja OR ‘khmer republic’ OR Cambodian* OR Cameroon* OR cameroun* OR ‘Central African Republic’ OR ‘Centrafrican Republic’ OR Centrafrique OR ‘Central African Empire’ OR ‘Central African*’ OR Chad OR Tchad OR Chadian* OR China OR Beijing OR Chinese* OR Colombia* OR Columbia* OR Comoros OR ‘comoro islands’ OR Congo OR Congolese* OR Costa Rica* OR ‘Ivory Coast’ OR Ivorian* OR Cuba* OR Djibouti* OR Dominica* OR Egypt* OR ‘united arab republic’ OR ‘El Salvador’ OR Salvador* OR Eritrea* OR Eswatini OR Swaziland OR Swazi* OR Ethiopia* OR Gabon* OR Gaboon OR Gambia* OR ‘Georgia republic’ OR Georgian* OR Ghana* OR Grenada OR Grenadian* OR Guatemala* OR Guinea* OR Guinea-Bissau OR Guyana OR Guyanese OR Haiti* OR Honduras OR Honduran* OR India OR ‘Indian Union’ OR Indonesia* OR Iran OR Persia* OR Iranian* OR Iraq* OR Jamaica* OR Jordan* OR Kazakhstan* OR Kazak OR Kenya* OR Kenian* OR Kiribati* OR ‘i-kiribati’ OR Korea* OR Kosovo* OR Kosovar* OR Kyrgyz OR Kyrgyzstan OR Kirghiz* OR Kirgiz* OR Laos OR Laotian* OR Lebanon OR Lebanese* OR Lesotho OR Basutoland OR Mosotho* OR Basotho OR Basuto OR Sotho* OR ‘Liberia Sudan’ OR Libya OR Libyan* OR Madagascar OR ‘Malagasy Republic’ OR Madagascan* OR Malagasy OR Malawi* OR Malaysia* OR Malaya OR ‘North Borneo’ OR Maldives OR Maldivian OR Dhivehin OR Mali OR Malian* OR ‘Marshall Islands’ OR Marshallese* OR Mauritania* OR mauretania OR Micronesia* OR Mongolia* OR Morocco OR Moroccan* OR Mozambique OR mocambique OR Mozambican* OR Myanmar OR Burma OR Burmese* OR Myanmarese* OR Myanmese* OR Namibia* OR ‘southwest africa’ OR Nepal* OR Nicaragua* OR Niger* OR Nigeria* OR Macedonia* OR ‘former yugoslav republic’ OR Pakistan* OR Panama* OR ‘Papua New Guinea*’ OR Paraguay* OR Peru* OR Philippine* OR Filipin* OR Romania* OR Rumania* OR ‘Russian Federation’ OR Russia* OR Siberia OR Rwanda* OR Samoa* OR ‘Sao Tome*’ OR Santomean* OR Senegal* OR Serbia* OR ‘Sierra Leone*’ OR ‘Solomon Island*’ OR Somali* OR ‘South Africa*’ OR ‘South Sudan*’ OR ‘Sri Lanka*’ OR ceylon OR ‘Saint Lucia*’ OR ‘St. Lucia*’ OR ‘Saint Vincent and the Grenadines’ OR Vincentians OR Suriname* OR Surinam OR ‘Syrian Arab Republic’ OR Syrian* OR Tajikistan OR Tadzhik OR Tadzhikistan OR Tajik* OR Tanzania OR Tanganyika OR Zanzibar OR Tanzanian* OR Thailand OR siam OR Thai OR Thais OR ‘Timor-Leste’ OR Timor OR Timorese* OR Togo OR Togolese* OR Tonga OR Tongan* OR Batonga* OR Tunisia OR tunesia OR Tunisian* OR Turkey OR Turk* OR Istanbul OR Uganda* OR Ukraine OR Ukraina OR Ukrainian* OR Uzbekistan OR Uzbek* OR Vanuatu* OR ‘Ni-Vanuatu’ OR ‘Ni-Van’ OR Vietnam OR ‘Viet Nam’ OR Vietnamese* OR ‘West Bank’ OR Gaza OR Yemen OR Aden OR Yemeni* OR Zambia* OR ‘northern rhodesia’ OR Zimbabwe OR Rhodesia OR Zimbabwean* OR ‘Dominican Republic’ OR Dominican* OR Mauritius OR Mauritian* OR Turkmenistan OR Turkmen* OR Turkmenian* OR ‘Equatorial Guinea’ OR Mexico OR Mexican* OR Tuvalu OR Tuvaluan* OR Ecuador* OR Moldova OR Moldavia OR Moldavian* OR Fiji* OR Montenegro OR Montenegrin*):ti,ab,kw OR (‘Sao Tome’ NEAR/1 Principe):ti,ab,kw)  AND  ('outpatient department'/exp OR 'health personnel attitude'/exp OR 'clinician'/exp OR 'doctor patient relationship'/exp OR 'professional-patient relationship'/exp OR 'nurse patient relationship'/exp OR 'health care delivery'/exp OR 'health care facility'/exp OR 'health care personnel'/exp OR 'health center'/exp OR 'health service'/exp OR 'health systems'/exp OR 'patient care'/exp OR 'patient provider communication'/exp OR 'primary health care'/exp OR ((‘ambulatory care’ OR ambulant OR outpatient OR ‘out-patient’ OR ‘out patient’) NEAR/3 (facility* OR ‘operation room’ OR care OR department* OR clinic* OR unit*)):ti,ab,kw OR (day NEAR/2 (clinic OR clinics OR hospital*)):ti,ab,kw OR (policlinic OR polyclinic OR surgicenters OR ‘clinical care’ OR clinician* OR ‘patient provider*’):ti,ab,kw OR (attitude NEAR/3 (‘health personnel’ OR ‘health care personnel’ OR ‘healthcare personnel’)):ti,ab,kw OR ((‘clinician-patient’ OR ‘doctor patient’ OR ‘patient doctor’ OR ‘hospital patient’ OR ‘patient physician’ OR ‘physician patient’ OR ‘physician-patient’ OR ‘patient staff’ OR ‘patient therapist’ OR therapeutic OR ‘therapist patient’ OR ‘professional-patient’ OR ‘patient-health’ OR ‘patient-healthcare’ OR ‘nurse patient’ OR ‘nurse-patient’ OR ‘patient-nurse’) NEAR/3 (relation* OR contact)):ti,ab,kw OR (‘bedside psychology’):ti,ab,kw OR ((health OR healthcare) NEAR/3 (delivery OR facility*)):ti,ab,kw OR ((health OR healthcare) NEAR/2 (personnel OR practitioner* OR professional* OR provider* OR worker* OR aid*)):ti,ab,kw OR ((health OR healthcare OR physician) NEAR/3 (center* OR centre* OR clinic OR clinics OR institute* OR service* OR agenc* OR setting*)):ti,ab,kw OR ((patient OR continuity OR episode* OR ‘patient-centered’ OR ‘Patient-Centred’ OR ‘patient centered’ OR ‘patient centred’ OR ‘person centered’ OR ‘person centred’ OR ‘person-centered’ OR ‘person-centred’) NEAR/3 (care OR management)):ti,ab,kw OR ((primary OR ‘first line’) NEAR/3 (care OR caregivers OR healthcare)):ti,ab,kw OR (provider NEAR/2 (attitude* OR service*)):ti,ab,kw)  AND  ('discrimination'/exp OR 'in service training'/exp OR 'interpersonal communication'/exp OR 'mentor'/exp OR 'mentoring'/exp OR 'patient engagement'/exp OR 'patient centered communication'/exp OR 'patient centeredness'/exp OR 'total quality management'/exp OR 'relationships'/exp OR 'root cause analysis'/exp OR 'sensitivity training'/exp OR 'social responsibility'/exp OR 'team based learning'/exp OR 'training'/exp OR ((Childcentered OR ‘child centered care’ OR clientcentered OR ‘client centered’ OR responsive) NEAR/3 (care OR ‘health service*’ OR healthservice*)):ti,ab,kw OR ((‘in service’ OR inservice OR ‘in-service’ OR rights) NEAR/3 training):ti,ab,kw OR ((judgmental OR discriminatory) NEAR/2 attitude*):ti,ab,kw OR ((quality OR QI) NEAR/3 (improvement OR management OR approach)):ti,ab,kw OR (social NEAR/3 (accountability OR responsibility* OR obligation*)):ti,ab,kw OR (‘Community Score Card’ OR discrimination OR ‘enhanced patient care’ OR communication OR disclosure OR ‘teach-back’ OR KidzAlive OR ‘KidzAlive-trained’ OR mentor* OR ‘patient engagement’ OR ‘patient-centered’ OR ‘patient centered’ OR ‘patient-centred’ OR ‘patient centred’ OR ‘provider training’ OR ‘root cause analysis’ OR ‘root cause analyses’ OR ‘sensitisation training*’ OR ‘sensitivity training*’ OR ‘Team-based learning’ OR ‘team based learning’ OR ‘tests of change’):ti,ab,kw)  AND  ('patient'/exp OR 'men who have sex with men'/exp OR 'sex worker'/exp OR 'prostitution'/exp OR 'LGBTQIA+ people'/exp OR (men NEAR/3 'sex with men'):ti,ab,kw OR (callgirl* OR prostitute* OR 'sex work*' OR prostitution):ti,ab,kw OR ((HIV OR HIV+ OR ‘Human immunodeficiency virus’) NEAR/3 (child OR children OR adolescent* OR teen OR teenager OR youth* OR pregnancy OR pregnant OR breastfeed* OR ‘breast feed*’)):ti,ab,kw OR patient*:ti,ab,kw OR (LGBTQIA+ OR Bisexual* OR gay* OR lesbian* OR pansexual* OR queer* OR asexual* OR transgender* OR intersex* OR ‘gender-expansive’ OR questioning OR GLBTI* OR GLBTQ* OR trans OR genderqueer OR ‘two-spirit*’ OR ‘two spirit*’ ‘2 spirit*’ OR ‘2-spirit*’ OR transsexual OR ‘gender diverse’ OR LGBTI* OR LGBTQ* OR LGBTT*):ti,ab,kw) |
| 10.1.23 | Ovid Medline | (exp HIV Infections/ OR exp HIV/ OR exp HIV-1/ OR exp HIV-2/ OR exp Antiretroviral Therapy, Highly Active/ OR exp Anti-Retroviral Agents/ OR exp Anti-HIV Agents/ OR (Human immunodeficiency virus OR HIV OR HIV-related OR HIV-positive OR HIV+).ti,ab,kf. OR ((aids associated OR aids related) ADJ3 (lentivirus OR retrovirus OR virus)).ti,ab,kf. OR ((human immuno deficiency OR immunodeficiency associated OR lymphadenopathy associated) ADJ3 (virus OR retrovirus)).ti,ab,kf. OR ((antiretroviral OR anti-retroviral OR Anti-HIV) ADJ3 (therapy OR agent*)).ti,ab,kf.)  AND  (exp Afghanistan/ OR exp Albania/ OR exp Algeria/ OR exp American Samoa/ OR exp Angola/ OR exp Argentina/ OR exp Armenia/ OR exp Azerbaijan/ OR exp Bangladesh/ OR exp "Republic of Belarus"/ OR exp Belize/ OR exp Benin/ OR exp Bhutan/ OR exp Bolivia/ OR exp "Bosnia and Herzegovina"/ OR exp Botswana/ OR exp Brazil/ OR exp Bulgaria/ OR exp Burkina Faso/ OR exp Burundi/ OR Cabo Verde/ OR exp Cambodia/ OR exp Cameroon/ OR exp Central African Republic/ OR exp Chad/ OR exp China/ OR exp Colombia/ OR exp Comoros/ OR exp Congo/ OR exp "Democratic Republic of the Congo"/ OR exp Costa Rica/ OR exp Cote d'Ivoire/ OR exp Cuba/ OR exp Djibouti/ OR exp Dominica/ OR exp Dominican Republic/ OR exp Egypt/ OR exp El Salvador/ OR exp Eritrea/ OR exp Eswatini/ OR exp Ethiopia/ OR exp Gabon/ OR exp Gambia/ OR exp "Georgia (Republic)"/ OR exp Ghana/ OR exp Grenada/ OR exp Guatemala/ OR 'Guinea-Bissau' OR exp Guyana/ OR exp Haiti/ OR exp Honduras/ OR exp India/ OR exp Indonesia/ OR exp Iran/ OR exp Iraq/ OR exp Jamaica/ OR exp Jordan/ OR exp Kazakhstan/ OR exp Kenya/ OR exp "Republic of Korea"/ OR exp "Democratic People's Republic of Korea"/ OR exp Korea/ OR exp Kosovo/ OR exp Kyrgyzstan/ OR exp Laos/ OR exp Lebanon/ OR exp Lesotho/ OR exp Libya/ OR exp Madagascar/ OR exp Malawi/ OR exp Malaysia/ OR exp Indian Ocean Islands/ OR exp Micronesia/ OR exp Mauritania/ OR exp Mongolia/ OR exp Morocco/ OR exp Mozambique/ OR exp Myanmar/ OR exp Namibia/ OR exp Nepal/ OR exp Nicaragua/ OR exp Niger/ OR exp Nigeria/ OR exp "Republic of North Macedonia"/ OR exp Pakistan/ OR exp Panama/ OR exp Papua New Guinea/ OR exp Paraguay/ OR exp Peru/ OR exp Philippines/ OR exp Romania/ OR exp Russia/ OR exp Rwanda/ OR exp Samoa/ OR exp "Sao Tome and Principe"/ OR exp Senegal/ OR exp Serbia/ OR exp Sierra Leone/ OR exp Somalia/ OR exp South Africa/ OR exp South Sudan/ OR 'Sri Lanka'/ OR exp Saint Lucia/ OR exp "Saint Vincent and the Grenadines"/ OR exp Suriname/ OR exp Syria/ OR exp Tajikistan/ OR exp Tanzania/ OR exp Thailand/ OR exp Timor-Leste/ OR exp Togo/ OR exp Tonga/ OR exp Tunisia/ OR exp Turkey/ OR exp Uganda/ OR exp Ukraine/ OR exp Uzbekistan/ OR exp Vanuatu/ OR exp Vietnam/ OR exp Yemen/ OR exp Zambia/ OR exp Zimbabwe/ OR exp Dominican Republic/ OR exp Mauritius/ OR exp Turkmenistan/ OR exp Equatorial Guinea/ OR exp Mexico/ OR exp Ecuador/ OR exp Moldova/ OR exp Fiji/ OR exp Montenegro/ OR (Afghanistan OR Afghan OR Afghans OR Albania* OR Algeria* OR American Samoa* OR Angola* OR Argentina OR Argentinian* OR Armenia* OR Azerbaijan* OR Bangladesh* OR Belarus* OR Belize* OR Benin* OR Bhutan* OR Bolivia* OR Bosnia and Herzegovina OR Bosnian* OR Bosniak* OR Botswana OR Bechuanaland OR Brazil OR Brazilian* OR Bulgaria* OR Burkina Faso OR upper volta OR Burundi OR Cabo Verde OR Cape Verde OR Cambodia OR kampudja OR khmer republic OR Cambodian* OR Cameroon* OR cameroun* OR Central African Republic OR Centrafrican Republic OR Centrafrique OR Central African Empire OR Central African* OR Chad OR Tchad OR Chadian* OR China OR Beijing OR Chinese* OR Colombia* OR Columbia* OR Comoros OR comoro islands OR Congo OR Congolese* OR Costa Rica* OR Ivory Coast OR Ivorian* OR Cuba* OR Djibouti* OR Dominica* OR Egypt* OR united arab republic OR El Salvador OR Salvador* OR Eritrea* OR Eswatini OR Swaziland OR Swazi* OR Ethiopia* OR Gabon* OR Gaboon OR Gambia* OR Georgia republic OR Georgian* OR Ghana* OR Grenada OR Grenadian* OR Guatemala* OR Guinea* OR Guinea-Bissau OR Guyana OR Guyanese OR Haiti* OR Honduras OR Honduran* OR India OR Indian Union OR Indonesia* OR Iran OR Persia* OR Iranian* OR Iraq* OR Jamaica* OR Jordan* OR Kazakhstan* OR Kazak OR Kenya* OR Kenian* OR Kiribati* OR i-kiribati OR Korea* OR Kosovo* OR Kosovar* OR Kyrgyz OR Kyrgyzstan OR Kirghiz* OR Kirgiz* OR Laos OR Laotian* OR Lebanon OR Lebanese* OR Lesotho OR Basutoland OR Mosotho* OR Basotho OR Basuto OR Sotho* OR Liberia Sudan OR Libya OR Libyan* OR Madagascar OR Malagasy Republic OR Madagascan* OR Malagasy OR Malawi* OR Malaysia* OR Malaya OR North Borneo OR Maldives OR Maldivian OR Dhivehin OR Mali OR Malian* OR Marshall Islands OR Marshallese* OR Mauritania* OR mauretania OR Micronesia* OR Mongolia* OR Morocco OR Moroccan* OR Mozambique OR mocambique OR Mozambican* OR Myanmar OR Burma OR Burmese* OR Myanmarese* OR Myanmese* OR Namibia* OR southwest africa OR Nepal* OR Nicaragua* OR Niger* OR Nigeria* OR Macedonia* OR former yugoslav republic OR Pakistan* OR Panama* OR Papua New Guinea* OR Paraguay* OR Peru* OR Philippine* OR Filipin* OR Romania* OR Rumania* OR Russian Federation OR Russia* OR Siberia OR Rwanda* OR Samoa* OR Sao Tome* OR Santomean* OR Senegal* OR Serbia* OR Sierra Leone* OR Solomon Island* OR Somali* OR South Africa* OR South Sudan* OR Sri Lanka* OR ceylon OR Saint Lucia* OR St Lucia* OR Saint Vincent and the Grenadines OR Vincentians OR Suriname* OR Surinam OR Syrian Arab Republic OR Syrian* OR Tajikistan OR Tadzhik OR Tadzhikistan OR Tajik* OR Tanzania OR Tanganyika OR Zanzibar OR Tanzanian* OR Thailand OR siam OR Thai OR Thais OR Timor-Leste OR Timor OR Timorese* OR Togo OR Togolese* OR Tonga OR Tongan* OR Batonga* OR Tunisia OR tunesia OR Tunisian* OR Turkey OR Turk* OR Istanbul OR Uganda* OR Ukraine OR Ukraina OR Ukrainian* OR Uzbekistan OR Uzbek* OR Vanuatu* OR Ni-Vanuatu OR Ni-Van OR Vietnam OR Viet Nam OR Vietnamese* OR West Bank OR Gaza OR Yemen OR Aden OR Yemeni* OR Zambia* OR northern rhodesia OR Zimbabwe OR Rhodesia OR Zimbabwean* OR Dominican Republic OR Dominican* OR Mauritius OR Mauritian* OR Turkmenistan OR Turkmen* OR Turkmenian* OR Equatorial Guinea OR Mexico OR Mexican* OR Tuvalu OR Tuvaluan* OR Ecuador* OR Moldova OR Moldavia OR Moldavian* OR Fiji* OR Montenegro OR Montenegrin*).ti,ab,kf. OR (Sao Tome ADJ1 Principe).ti,ab,kf.)  AND  (exp Outpatient Clinics, Hospital/ OR exp "Attitude of Health Personnel"/ OR exp Nurse Clinicians/ OR exp Physician-Patient Relations/ OR exp Professional-Patient Relations/ OR exp Nurse-Patient Relations/ OR exp "Delivery of Health Care"/ OR exp Health Facilities/ OR exp Health Personnel/ OR exp Health Services/ OR exp Patient Care/ OR exp Primary Health Care/ OR ((ambulatory care OR ambulant OR outpatient OR out-patient OR out patient) ADJ3 (facility* OR operation room OR care OR department* OR clinic* OR unit*)).ti,ab,kf. OR (day ADJ2 (clinic OR clinics OR hospital*)).ti,ab,kf. OR (policlinic OR polyclinic OR surgicenters OR clinical care OR clinician* OR patient provider*).ti,ab,kf. OR (attitude ADJ3 (health personnel OR health care personnel OR healthcare personnel)).ti,ab,kf. OR ((clinician-patient OR doctor patient OR patient doctor OR hospital patient OR patient physician OR physician patient OR physician-patient OR patient staff OR patient therapist OR therapeutic OR therapist patient OR professional-patient OR patient-health OR patient-healthcare OR nurse patient OR nurse-patient OR patient-nurse) ADJ3 (relation* OR contact)).ti,ab,kf. OR (bedside psychology).ti,ab,kf. OR ((health OR healthcare) ADJ3 (delivery OR facility*)).ti,ab,kf. OR ((health OR healthcare) ADJ2 (personnel OR practitioner* OR professional* OR provider* OR worker* OR aid*)).ti,ab,kf. OR ((health OR healthcare OR physician) ADJ3 (center* OR centre* OR clinic OR clinics OR institute* OR service* OR agenc* OR setting*)).ti,ab,kf. OR ((patient OR continuity OR episode* OR patient-centered) ADJ3 (care OR management)).ti,ab,kf. OR ((primary OR first line) ADJ3 (care OR caregivers OR healthcare)).ti,ab,kf. OR (provider ADJ2 (attitude* OR service*)).ti,ab,kf.)  AND  (exp Social Discrimination/ OR exp Inservice Training/ OR exp Communication/ OR exp Mentors/ OR exp Mentoring/ OR exp Patient Participation/ OR exp Patient-Centered Care/ OR exp Total Quality Management/ OR exp "Root Cause Analysis"/ OR exp Sensitivity Training Groups/ OR exp Social Responsibility/ OR exp Problem-Based Learning/ OR ((Childcentered OR child centered care OR clientcentered OR client centered OR responsive) ADJ3 (care OR health service* OR healthservice*)).ti,ab,kf. OR ((in service OR inservice OR in-service OR rights) ADJ3 training).ti,ab,kf. OR ((judgmental OR discriminatory) ADJ2 attitude*).ti,ab,kf. OR ((quality OR QI) ADJ3 (improvement OR management OR approach)).ti,ab,kf. OR (social ADJ3 (accountability OR responsibility* OR obligation*)).ti,ab,kf. OR (Community Score Card OR discrimination OR enhanced patient care OR communication OR disclosure OR teach-back OR KidzAlive OR KidzAlive-trained OR mentor* OR patient engagement OR patient-centered OR patient centered OR Patient-Centred OR patient centered OR patient centred OR person centered OR person centred OR person-centered OR person-centred OR provider training OR root cause analysis OR root cause analyses OR sensitisation training* OR sensitivity training* OR Team-based learning OR team based learning OR tests of change).ti,ab,kf.)  AND  (exp Patients/ OR exp "Sexual and Gender Minorities"/ OR exp Sex Workers/ OR exp Sex Work/ OR (men ADJ3 sex with men).ti,ab,kf. OR (callgirl* OR prostitute* OR sex work* OR prostitution).ti,ab,kf. OR ((HIV OR HIV+ OR Human immunodeficiency virus) ADJ3 (child OR children OR adolescent* OR teen OR teenager OR youth* OR pregnancy OR pregnant OR breastfeed* OR breast feed*)).ti,ab,kf. OR patient*.ti,ab,kf. OR (LGBTQIA+ OR Bisexual* OR gay* OR lesbian* OR pansexual* OR queer* OR asexual* OR transgender* OR hiv-related OR intersex* OR gender-expansive OR questioning OR GLBTI* OR GLBTQ* OR trans OR genderqueer OR two-spirit* OR two spirit* 2 spirit* OR 2-spirit* OR transsexual OR gender diverse OR LGBTI* OR LGBTQ* OR LGBTT*).ti,ab,kf.) |
| 10.1.23 | SCOPUS | ((TITLE-ABS-KEY(“Human immunodeficiency virus” OR HIV OR “HIV-related” OR “HIV-positive” OR HIV+)) OR (TITLE-ABS-KEY((“aids associated” OR “aids related”) W/3 (lentivirus OR retrovirus OR virus))) OR (TITLE-ABS-KEY((“human immuno deficiency” OR “immunodeficiency associated” OR “lymphadenopathy associated”) W/3 (virus OR retrovirus))) OR (TITLE-ABS-KEY((antiretroviral OR “anti-retroviral” OR “Anti-HIV”) W/3 (therapy OR agent*))))  AND  ((TITLE-ABS-KEY(Afghanistan OR Afghan OR Afghans OR Albania* OR Algeria* OR “American Samoa*” OR Angola* OR Argentina OR Argentinian* OR Armenia* OR Azerbaijan* OR Bangladesh* OR Belarus* OR Belize* OR Benin* OR Bhutan* OR Bolivia* OR “Bosnia and Herzegovina” OR Bosnian* OR Bosniak* OR Botswana OR Bechuanaland OR Brazil OR Brazilian* OR Bulgaria* OR “Burkina Faso” OR “upper volta” OR Burundi OR “Cabo Verde” OR “Cape Verde” OR Cambodia OR kampudja OR “khmer republic” OR Cambodian* OR Cameroon* OR cameroun* OR “Central African Republic” OR “Centrafrican Republic” OR Centrafrique OR “Central African Empire” OR “Central African*” OR Chad OR Tchad OR Chadian* OR China OR Beijing OR Chinese* OR Colombia* OR Columbia* OR Comoros OR “comoro islands” OR Congo OR Congolese* OR Costa Rica* OR “Ivory Coast” OR Ivorian* OR Cuba* OR Djibouti* OR Dominica* OR Egypt* OR “united arab republic” OR “El Salvador” OR Salvador* OR Eritrea* OR Eswatini OR Swaziland OR Swazi* OR Ethiopia* OR Gabon* OR Gaboon OR Gambia* OR “Georgia republic” OR Georgian* OR Ghana* OR Grenada OR Grenadian* OR Guatemala* OR Guinea* OR Guinea-Bissau OR Guyana OR Guyanese OR Haiti* OR Honduras OR Honduran* OR India OR “Indian Union” OR Indonesia* OR Iran OR Persia* OR Iranian* OR Iraq* OR Jamaica* OR Jordan* OR Kazakhstan* OR Kazak OR Kenya* OR Kenian* OR Kiribati* OR “i-kiribati” OR Korea* OR Kosovo* OR Kosovar* OR Kyrgyz OR Kyrgyzstan OR Kirghiz* OR Kirgiz* OR Laos OR Laotian* OR Lebanon OR Lebanese* OR Lesotho OR Basutoland OR Mosotho* OR Basotho OR Basuto OR Sotho* OR “Liberia Sudan” OR Libya OR Libyan* OR Madagascar OR “Malagasy Republic” OR Madagascan* OR Malagasy OR Malawi* OR Malaysia* OR Malaya OR “North Borneo” OR Maldives OR Maldivian OR Dhivehin OR Mali OR Malian* OR “Marshall Islands” OR Marshallese* OR Mauritania* OR mauretania OR Micronesia* OR Mongolia* OR Morocco OR Moroccan* OR Mozambique OR mocambique OR Mozambican* OR Myanmar OR Burma OR Burmese* OR Myanmarese* OR Myanmese* OR Namibia* OR “southwest africa” OR Nepal* OR Nicaragua* OR Niger* OR Nigeria* OR Macedonia* OR “former yugoslav republic” OR Pakistan* OR Panama* OR “Papua New Guinea*” OR Paraguay* OR Peru* OR Philippine* OR Filipin* OR Romania* OR Rumania* OR “Russian Federation” OR Russia* OR Siberia OR Rwanda* OR Samoa* OR “Sao Tome*” OR Santomean* OR Senegal* OR Serbia* OR “Sierra Leone*” OR “Solomon Island*” OR Somali* OR “South Africa*” OR “South Sudan*” OR “Sri Lanka*” OR ceylon OR “Saint Lucia*” OR “St. Lucia*” OR “Saint Vincent and the Grenadines” OR Vincentians OR Suriname* OR Surinam OR “Syrian Arab Republic” OR Syrian* OR Tajikistan OR Tadzhik OR Tadzhikistan OR Tajik* OR Tanzania OR Tanganyika OR Zanzibar OR Tanzanian* OR Thailand OR siam OR Thai OR Thais OR “Timor-Leste” OR Timor OR Timorese* OR Togo OR Togolese* OR Tonga OR Tongan* OR Batonga* OR Tunisia OR tunesia OR Tunisian* OR Turkey OR Turk* OR Istanbul OR Uganda* OR Ukraine OR Ukraina OR Ukrainian* OR Uzbekistan OR Uzbek* OR Vanuatu* OR “Ni-Vanuatu” OR “Ni-Van” OR Vietnam OR “Viet Nam” OR Vietnamese* OR “West Bank” OR Gaza OR Yemen OR Aden OR Yemeni* OR Zambia* OR “northern rhodesia” OR Zimbabwe OR Rhodesia OR Zimbabwean* OR “Dominican Republic” OR Dominican* OR Mauritius OR Mauritian* OR Turkmenistan OR Turkmen* OR Turkmenian* OR “Equatorial Guinea” OR Mexico OR Mexican* OR Tuvalu OR Tuvaluan* OR Ecuador* OR Moldova OR Moldavia OR Moldavian* OR Fiji* OR Montenegro OR Montenegrin*)) OR (TITLE-ABS-KEY(“Sao Tome” W/1 Principe)))  AND ((TITLE-ABS-KEY((“ambulatory care” OR ambulant OR outpatient OR “out-patient” OR “out patient”) W/3 (facility* OR “operation room” OR care OR department* OR clinic* OR unit*))) OR (TITLE-ABS-KEY(day W/2 (clinic OR clinics OR hospital*))) OR (TITLE-ABS-KEY(policlinic OR polyclinic OR surgicenters OR “clinical care” OR clinician* OR “patient provider*”)) OR (TITLE-ABS-KEY(attitude W/3 (“health personnel” OR “health care personnel” OR “healthcare personnel”))) OR (TITLE-ABS-KEY((“clinician-patient” OR “doctor patient” OR “patient doctor” OR “hospital patient” OR “patient physician” OR “physician patient” OR “physician-patient” OR “patient staff” OR “patient therapist” OR therapeutic OR “therapist patient” OR “professional-patient” OR “patient-health” OR “patient-healthcare” OR “nurse patient” OR “nurse-patient” OR “patient-nurse”) W/3 (relation* OR contact))) OR (TITLE-ABS-KEY(“bedside psychology”)) OR (TITLE-ABS-KEY((health OR healthcare) W/3 (delivery OR facility*))) OR (TITLE-ABS-KEY((health OR healthcare) W/2 (personnel OR practitioner* OR professional* OR provider* OR worker* OR aid*))) OR (TITLE-ABS-KEY((health OR healthcare OR physician) W/3 (center* OR centre* OR clinic OR clinics OR institute* OR service* OR agenc* OR setting*))) OR (TITLE-ABS-KEY((patient OR continuity OR episode* OR “patient-centered”) W/3 (care OR management))) OR (TITLE-ABS-KEY((primary OR “first line”) W/3 (care OR caregivers OR healthcare))) OR (TITLE-ABS-KEY(provider W/2 (attitude* OR service*))))  AND  ((TITLE-ABS-KEY((Childcentered OR “child centered care” OR clientcentered OR “client centered” OR responsive) W/3 (care OR “health service*” OR healthservice*))) OR (TITLE-ABS-KEY((“in service” OR inservice OR “in-service” OR rights) W/3 training)) OR (TITLE-ABS-KEY((judgmental OR discriminatory) W/2 attitude*)) OR (TITLE-ABS-KEY((quality OR QI) W/3 (improvement OR management OR approach))) OR (TITLE-ABS-KEY(social W/3 (accountability OR responsibility* OR obligation*))) OR (TITLE-ABS-KEY(“Community Score Card” OR discrimination OR “enhanced patient care” OR communication OR disclosure OR “teach-back” OR KidzAlive OR “KidzAlive-trained” OR mentor* OR “patient engagement” OR “patient-centered” OR “patient centered” OR “Patient-Centred” OR “patient centered” OR “patient centred” OR “person centered” OR “person centred” OR “person-centered” OR “person-centred” OR “provider training” OR “root cause analysis” OR “root cause analyses” OR “sensitisation training*” OR “sensitivity training*” OR “Team-based learning” OR “team based learning” OR “tests of change”)))  AND  ((TITLE-ABS-KEY(men W/3 “sex with men”)) OR (TITLE-ABS-KEY(callgirl* OR prostitute* OR “sex work*” OR prostitution)) OR (TITLE-ABS-KEY((HIV OR HIV+ OR “Human immunodeficiency virus”) W/3 (child OR children OR adolescent* OR teen OR teenager OR youth* OR pregnancy OR pregnant OR breastfeed* OR “breast feed*”))) OR (TITLE-ABS-KEY(patient*)) OR (TITLE-ABS-KEY(LGBTQIA+ OR Bisexual* OR gay* OR lesbian* OR pansexual* OR queer* OR asexual* OR transgender* OR intersex* OR “gender-expansive” OR questioning OR GLBTI* OR GLBTQ* OR trans OR genderqueer OR “two-spirit*” OR “two spirit*” “2 spirit*” OR “2-spirit*” OR transsexual OR “gender diverse” OR LGBTI* OR LGBTQ* OR LGBTT*))) |
| 10.1.23 | The Cochrane library | ([mh “HIV Infections”] OR [mh “HIV”] OR [mh “HIV-1”] OR [mh “HIV-2”] OR [mh “Antiretroviral Therapy, Highly Active”] OR [mh “Anti-Retroviral Agents”] OR [mh “Anti-HIV Agents”] OR (“Human immunodeficiency virus” OR HIV OR “HIV related” OR “HIV positive”):ti,ab,kw OR ((“aids associated” OR “aids related”) NEAR/3 (lentivirus OR retrovirus OR virus)):ti,ab,kw OR ((“human immuno deficiency” OR “immunodeficiency associated” OR “lymphadenopathy associated”) NEAR/3 (virus OR retrovirus)):ti,ab,kw OR ((antiretroviral OR “anti retroviral” OR “Anti HIV”) NEAR/3 (therapy OR agent*)):ti,ab,kw)  AND  ([mh “Afghanistan”] OR [mh “Albania”] OR [mh “Algeria”] OR [mh “American Samoa”] OR [mh “Angola”] OR [mh “Argentina”] OR [mh “Armenia”] OR [mh “Azerbaijan”] OR [mh “Bangladesh”] OR [mh “Republic of Belarus”] OR [mh “Belize”] OR [mh “Benin”] OR [mh “Bhutan”] OR [mh “Bolivia”] OR [mh “Bosnia and Herzegovina”] OR [mh “Botswana”] OR [mh “Brazil”] OR [mh “Bulgaria”] OR [mh “Burkina Faso”] OR [mh “Burundi”] OR [mh “Cambodia”] OR [mh “Cameroon”] OR [mh “Central African Republic”] OR [mh “Chad”] OR [mh “China”] OR [mh “Colombia”] OR [mh “Comoros”] OR [mh “Congo”] OR [mh “Costa Rica”] OR [mh “Cote d Ivoire”] OR [mh “Cuba”] OR [mh “Djibouti”] OR [mh “Dominica”] OR [mh “Dominican Republic”] OR [mh “Egypt”] OR [mh “El Salvador”] OR [mh “Eritrea”] OR [mh “Eswatini”] OR [mh “Ethiopia”] OR [mh “Gabon”] OR [mh “Gambia”] OR [mh “Georgia (Republic)”] OR [mh “Ghana”] OR [mh “Grenada”] OR [mh “Guatemala”] OR 'Guinea-Bissau' OR [mh “Guyana”] OR [mh “Haiti”] OR [mh “Honduras”] OR [mh “India”] OR [mh “Indonesia”] OR [mh “Iran”] OR [mh “Iraq”] OR [mh “Jamaica”] OR [mh “Jordan”] OR [mh “Kazakhstan”] OR [mh “Kenya”] OR [mh “Korea”] OR [mh “Kosovo”] OR [mh “Kyrgyzstan”] OR [mh “Laos”] OR [mh “Lebanon”] OR [mh “Lesotho”] OR [mh “Libya”] OR [mh “Madagascar”] OR [mh “Malawi”] OR [mh “Malaysia”] OR [mh “Indian Ocean Islands”] OR [mh “Micronesia”] OR [mh “Mauritania”] OR [mh “Mongolia”] OR [mh “Morocco”] OR [mh “Mozambique”] OR [mh “Myanmar”] OR [mh “Namibia”] OR [mh “Nepal”] OR [mh “Nicaragua”] OR [mh “Niger”] OR [mh “Nigeria”] OR [mh “Pakistan”] OR [mh “Panama”] OR [mh “Papua New Guinea”] OR [mh “Paraguay”] OR [mh “Peru”] OR [mh “Philippines”] OR [mh “Romania”] OR [mh “Russia”] OR [mh “Rwanda”] OR [mh “Samoa”] OR [mh “Senegal”] OR [mh “Serbia”] OR [mh “Sierra Leone”] OR [mh “Somalia”] OR [mh “South Africa”] OR [mh “South Sudan”] OR [mh “Sri Lanka”] OR [mh “Saint Lucia”] OR [mh “Suriname”] OR [mh “Syria”] OR [mh “Tajikistan”] OR [mh “Tanzania”] OR [mh “Thailand”] OR [mh “Timor Leste”] OR [mh “Togo”] OR [mh “Tonga”] OR [mh “Tunisia”] OR [mh “Turkey”] OR [mh “Uganda”] OR [mh “Ukraine”] OR [mh “Uzbekistan”] OR [mh “Vanuatu”] OR [mh “Vietnam”] OR [mh “Yemen”] OR [mh “Zambia”] OR [mh “Zimbabwe”] OR [mh “Dominican Republic”] OR [mh “Mauritius”] OR [mh “Turkmenistan”] OR [mh “Equatorial Guinea”] OR [mh “Mexico”] OR [mh “Ecuador”] OR [mh “Moldova”] OR [mh “Fiji”] OR [mh “Montenegro”] OR (Afghanistan OR Afghan OR Afghans OR Albania* OR Algeria* OR “American Samoa*” OR Angola* OR Argentina OR Argentinian* OR Armenia* OR Azerbaijan* OR Bangladesh* OR Belarus* OR Belize* OR Benin* OR Bhutan* OR Bolivia* OR “Bosnia and Herzegovina” OR Bosnian* OR Bosniak* OR Botswana OR Bechuanaland OR Brazil OR Brazilian* OR Bulgaria* OR “Burkina Faso” OR “upper volta” OR Burundi OR “Cabo Verde” OR “Cape Verde” OR Cambodia OR kampudja OR “khmer republic” OR Cambodian* OR Cameroon* OR cameroun* OR “Central African Republic” OR “Centrafrican Republic” OR Centrafrique OR “Central African Empire” OR “Central African*” OR Chad OR Tchad OR Chadian* OR China OR Beijing OR Chinese* OR Colombia* OR Columbia* OR Comoros OR “comoro islands” OR Congo OR Congolese* OR Costa Rica* OR “Ivory Coast” OR Ivorian* OR Cuba* OR Djibouti* OR Dominica* OR Egypt* OR “united arab republic” OR “El Salvador” OR Salvador* OR Eritrea* OR Eswatini OR Swaziland OR Swazi* OR Ethiopia* OR Gabon* OR Gaboon OR Gambia* OR “Georgia republic” OR Georgian* OR Ghana* OR Grenada OR Grenadian* OR Guatemala* OR Guinea* OR Guinea Bissau OR Guyana OR Guyanese OR Haiti* OR Honduras OR Honduran* OR India OR “Indian Union” OR Indonesia* OR Iran OR Persia* OR Iranian* OR Iraq* OR Jamaica* OR Jordan* OR Kazakhstan* OR Kazak OR Kenya* OR Kenian* OR Kiribati* OR “i kiribati” OR Korea* OR Kosovo* OR Kosovar* OR Kyrgyz OR Kyrgyzstan OR Kirghiz* OR Kirgiz* OR Laos OR Laotian* OR Lebanon OR Lebanese* OR Lesotho OR Basutoland OR Mosotho* OR Basotho OR Basuto OR Sotho* OR “Liberia Sudan” OR Libya OR Libyan* OR Madagascar OR “Malagasy Republic” OR Madagascan* OR Malagasy OR Malawi* OR Malaysia* OR Malaya OR “North Borneo” OR Maldives OR Maldivian OR Dhivehin OR Mali OR Malian* OR “Marshall Islands” OR Marshallese* OR Mauritania* OR mauretania OR Micronesia* OR Mongolia* OR Morocco OR Moroccan* OR Mozambique OR mocambique OR Mozambican* OR Myanmar OR Burma OR Burmese* OR Myanmarese* OR Myanmese* OR Namibia* OR “southwest africa” OR Nepal* OR Nicaragua* OR Niger* OR Nigeria* OR Macedonia* OR “former yugoslav republic” OR Pakistan* OR Panama* OR “Papua New Guinea*” OR Paraguay* OR Peru* OR Philippine* OR Filipin* OR Romania* OR Rumania* OR “Russian Federation” OR Russia* OR Siberia OR Rwanda* OR Samoa* OR “Sao Tome*” OR Santomean* OR Senegal* OR Serbia* OR “Sierra Leone*” OR “Solomon Island*” OR Somali* OR “South Africa*” OR “South Sudan*” OR “Sri Lanka*” OR ceylon OR “Saint Lucia*” OR “St. Lucia*” OR “Saint Vincent and the Grenadines” OR Vincentians OR Suriname* OR Surinam OR “Syrian Arab Republic” OR Syrian* OR Tajikistan OR Tadzhik OR Tadzhikistan OR Tajik* OR Tanzania OR Tanganyika OR Zanzibar OR Tanzanian* OR Thailand OR siam OR Thai OR Thais OR “Timor Leste” OR Timor OR Timorese* OR Togo OR Togolese* OR Tonga OR Tongan* OR Batonga* OR Tunisia OR tunesia OR Tunisian* OR Turkey OR Turk* OR Istanbul OR Uganda* OR Ukraine OR Ukraina OR Ukrainian* OR Uzbekistan OR Uzbek* OR Vanuatu* OR “Ni Vanuatu” OR “Ni Van” OR Vietnam OR “Viet Nam” OR Vietnamese* OR “West Bank” OR Gaza OR Yemen OR Aden OR Yemeni* OR Zambia* OR “northern rhodesia” OR Zimbabwe OR Rhodesia OR Zimbabwean* OR “Dominican Republic” OR Dominican* OR Mauritius OR Mauritian* OR Turkmenistan OR Turkmen* OR Turkmenian* OR “Equatorial Guinea” OR Mexico OR Mexican* OR Tuvalu OR Tuvaluan* OR Ecuador* OR Moldova OR Moldavia OR Moldavian* OR Fiji* OR Montenegro OR Montenegrin*):ti,ab,kw OR (“Sao Tome” NEAR/1 Principe):ti,ab,kw)  AND  ([mh “Outpatient Clinics, Hospital”] OR [mh “Attitude of Health Personnel”] OR [mh “Nurse Clinicians”] OR [mh “Physician-Patient Relations”] OR [mh “Professional-Patient Relations”] OR [mh “Nurse-Patient Relations”] OR [mh “Delivery of Health Care”] OR [mh “Health Facilities”] OR [mh “Health Personnel”] OR [mh “Health Services”] OR [mh “Patient Care”] OR [mh “Primary Health Care”] OR ((“ambulatory care” OR ambulant OR outpatient OR “out patient” OR “out patient”) NEAR/3 (facility* OR “operation room” OR care OR department* OR clinic* OR unit*)):ti,ab,kw OR (day NEAR/2 (clinic OR clinics OR hospital*)):ti,ab,kw OR (policlinic OR polyclinic OR surgicenters OR “clinical care” OR clinician* OR “patient provider*”):ti,ab,kw OR (attitude NEAR/3 (“health personnel” OR “health care personnel” OR “healthcare personnel”)):ti,ab,kw OR ((“clinician patient” OR “doctor patient” OR “patient doctor” OR “hospital patient” OR “patient physician” OR “physician patient” OR “physician patient” OR “patient staff” OR “patient therapist” OR therapeutic OR “therapist patient” OR “professional patient” OR “patient health” OR “patient healthcare” OR “nurse patient” OR “nurse patient” OR “patient nurse”) NEAR/3 (relation* OR contact)):ti,ab,kw OR (“bedside psychology”):ti,ab,kw OR ((health OR healthcare) NEAR/3 (delivery OR facility*)):ti,ab,kw OR ((health OR healthcare) NEAR/2 (personnel OR practitioner* OR professional* OR provider* OR worker* OR aid*)):ti,ab,kw OR ((health OR healthcare OR physician) NEAR/3 (center* OR centre* OR clinic OR clinics OR institute* OR service* OR agenc* OR setting*)):ti,ab,kw OR ((patient OR continuity OR episode* OR “patient centered”) NEAR/3 (care OR management)):ti,ab,kw OR ((primary OR “first line”) NEAR/3 (care OR caregivers OR healthcare)):ti,ab,kw OR (provider NEAR/2 (attitude* OR service*)):ti,ab,kw)  AND ([mh “Social Discrimination”] OR [mh “Inservice Training”] OR [mh “Communication”] OR [mh “Mentors”] OR [mh “Mentoring”] OR [mh “Patient Participation”] OR [mh “Patient Centered Care”] OR [mh “Total Quality Management”] OR [mh “Root Cause Analysis”] OR [mh “Sensitivity Training Groups”] OR [mh “Social Responsibility”] OR [mh “Problem Based Learning”] OR ((Childcentered OR “child centered care” OR clientcentered OR “client centered” OR responsive) NEAR/3 (care OR “health service*” OR healthservice*)):ti,ab,kw OR ((“in service” OR inservice OR “in service” OR rights) NEAR/3 training):ti,ab,kw OR ((judgmental OR discriminatory) NEAR/2 attitude*):ti,ab,kw OR ((quality OR QI) NEAR/3 (improvement OR management OR approach)):ti,ab,kw OR (social NEAR/3 (accountability OR responsibility* OR obligation*)):ti,ab,kw OR (“Community Score Card” OR discrimination OR “enhanced patient care” OR communication OR disclosure OR “teach back” OR KidzAlive OR “KidzAlive trained” OR mentor* OR “patient engagement” OR “patient centered” OR “patient centered” OR “Patient Centred” OR “patient centered” OR “patient centred” OR “person centered” OR “person centred” OR “person centered” OR “person centred” OR “provider training” OR “root cause analysis” OR “root cause analyses” OR “sensitisation training*” OR “sensitivity training*” OR “Team based learning” OR “team based learning” OR “tests of change”):ti,ab,kw)  AND ([mh “Patients”] OR [mh “Sexual and Gender Minorities”] OR [mh “Sex Workers”] OR [mh “Sex Work”] OR (men NEAR/3 “sex with men”):ti,ab,kw OR (callgirl* OR prostitute* OR “sex work*” OR prostitution):ti,ab,kw OR ((HIV OR “Human immunodeficiency virus”) NEAR/3 (child OR children OR adolescent* OR teen OR teenager OR youth* OR pregnancy OR pregnant OR breastfeed* OR “breast feed*”)):ti,ab,kw OR patient*:ti,ab,kw OR (LGBTQIA OR Bisexual* OR gay* OR lesbian* OR pansexual* OR queer* OR asexual* OR transgender* OR intersex* OR “gender expansive” OR questioning OR GLBTI* OR GLBTQ* OR trans OR genderqueer OR “two spirit*” OR “two spirit*” “2 spirit*” OR “2 spirit*” OR transsexual OR “gender diverse” OR LGBTI* OR LGBTQ* OR LGBTT*):ti,ab,kw) |
| 10.1.23 | CINAHL Plus | ((MH "Human Immunodeficiency Virus+") OR (MH "HIV-Positive Persons+") OR (MH "HIV-1") OR (MH "Antiretroviral Therapy, Highly Active") OR TI (“Human immunodeficiency virus” OR HIV OR “HIV-related” OR “HIV-positive” OR HIV+) OR TI ((“aids associated” OR “aids related”) N3 (lentivirus OR retrovirus OR virus)) OR TI ((“human immuno deficiency” OR “immunodeficiency associated” OR “lymphadenopathy associated”) N3 (virus OR retrovirus)) OR TI ((antiretroviral OR “anti-retroviral” OR “Anti-HIV”) N3 (therapy OR agent*)) OR AB (“Human immunodeficiency virus” OR HIV OR “HIV-related” OR “HIV-positive” OR HIV+) OR AB ((“aids associated” OR “aids related”) N3 (lentivirus OR retrovirus OR virus)) OR AB ((“human immuno deficiency” OR “immunodeficiency associated” OR “lymphadenopathy associated”) N3 (virus OR retrovirus)) OR AB ((antiretroviral OR “anti-retroviral” OR “Anti-HIV”) N3 (therapy OR agent*)))  AND ((MH "Afghanistan") OR (MH "Albania") OR (MH "Algeria") OR (MH "American Samoa") OR (MH "Angola") OR (MH "Argentina") OR (MH "Armenia") OR (MH "Azerbaijan") OR (MH "Bangladesh") OR (MH "Byelarus") OR (MH "Belize") OR (MH "Benin") OR (MH "Bhutan") OR (MH "Bolivia") OR (MH "Bosnia-Herzegovina") OR (MH "Botswana") OR (MH "Brazil") OR (MH "Bulgaria") OR (MH "Burkina Faso") OR (MH "Burundi") OR (MH "Cape Verde") OR (MH "Cambodia") OR (MH "Cameroon") OR (MH "Central African Republic") OR (MH "Chad") OR (MH "China") OR (MH "Colombia") OR (MH "Congo") OR (MH "Democratic Republic of the Congo") OR (MH "Costa Rica") OR (MH "Cote d'Ivoire") OR (MH "Cuba") OR (MH "Djibouti") OR (MH "Dominica") OR (MH "Dominican Republic") OR (MH "Egypt") OR (MH "El Salvador") OR (MH "Eritrea") OR (MH "Ethiopia") OR (MH "Gabon") OR (MH "Gambia") OR (MH "Georgia (Republic)") OR (MH "Ghana") OR (MH "Guatemala") OR (MH "Guinea-Bissau") OR (MH "Guyana") OR (MH "Haiti") OR (MH "Honduras") OR (MH "India") OR (MH "Indonesia") OR (MH "Iran") OR (MH "Iraq") OR (MH "Jamaica") OR (MH "Jordan") OR (MH "Kazakhstan") OR (MH "Kenya") OR (MH "South Korea") OR (MH "North Korea") OR (MH "Yugoslavia") OR (MH "Kyrgyzstan") OR (MH "Laos") OR (MH "Lebanon") OR (MH "Lesotho") OR (MH "Libya") OR (MH "Madagascar") OR (MH "Malawi") OR (MH "Malaysia") OR (MH "Indian Ocean Islands") OR (MH "Micronesia") OR (MH "Mauritania") OR (MH "Mongolia") OR (MH "Morocco") OR (MH "Mozambique") OR (MH "Myanmar") OR (MH "Namibia") OR (MH "Nepal") OR (MH "Nicaragua") OR (MH "Niger") OR (MH "Nigeria") OR (MH "Macedonia (Republic)") OR (MH "Pakistan") OR (MH "Panama") OR (MH "Papua New Guinea") OR (MH "Paraguay") OR (MH "Peru") OR (MH "Philippines") OR (MH "Romania") OR (MH "Russia") OR (MH "Rwanda") OR (MH "Samoa") OR (MH "Senegal") OR (MH "Serbia") OR (MH "Sierra Leone") OR (MH "Somalia") OR (MH "South Africa") OR (MH "Sudan") OR (MH "Sri Lanka") OR (MH "Suriname") OR (MH "Syria") OR (MH "Tajikistan") OR (MH "Tanzania") OR (MH "Thailand") OR (MH "Timor") OR (MH "East Timor") OR (MH "Togo") OR (MH "Polynesia") OR (MH "Tunisia") OR (MH "Turkey") OR (MH "Uganda") OR (MH "Ukraine") OR (MH "Uzbekistan") OR (MH "Vietnam") OR (MH "Yemen") OR (MH "Zambia") OR (MH "Zimbabwe") OR (MH "Dominican Republic") OR (MH "Indian Ocean Islands") OR (MH "Turkmenistan") OR (MH "Equatorial Guinea") OR (MH "Mexico") OR (MH "Ecuador") OR (MH "Moldova") OR (MH "Melanesia") OR TI (Afghanistan OR Afghan OR Afghans OR Albania* OR Algeria* OR “American Samoa*” OR Angola* OR Argentina OR Argentinian* OR Armenia* OR Azerbaijan* OR Bangladesh* OR Belarus* OR Belize* OR Benin* OR Bhutan* OR Bolivia* OR “Bosnia and Herzegovina” OR Bosnian* OR Bosniak* OR Botswana OR Bechuanaland OR Brazil OR Brazilian* OR Bulgaria* OR “Burkina Faso” OR “upper volta” OR Burundi OR “Cabo Verde” OR “Cape Verde” OR Cambodia OR kampudja OR “khmer republic” OR Cambodian* OR Cameroon* OR cameroun* OR “Central African Republic” OR “Centrafrican Republic” OR Centrafrique OR “Central African Empire” OR “Central African*” OR Chad OR Tchad OR Chadian* OR China OR Beijing OR Chinese* OR Colombia* OR Columbia* OR Comoros OR “comoro islands” OR Congo OR Congolese* OR Costa Rica* OR “Ivory Coast” OR Ivorian* OR Cuba* OR Djibouti* OR Dominica* OR Egypt* OR “united arab republic” OR “El Salvador” OR Salvador* OR Eritrea* OR Eswatini OR Swaziland OR Swazi* OR Ethiopia* OR Gabon* OR Gaboon OR Gambia* OR “Georgia republic” OR Georgian* OR Ghana* OR Grenada OR Grenadian* OR Guatemala* OR Guinea* OR Guinea-Bissau OR Guyana OR Guyanese OR Haiti* OR Honduras OR Honduran* OR India OR “Indian Union” OR Indonesia* OR Iran OR Persia* OR Iranian* OR Iraq* OR Jamaica* OR Jordan* OR Kazakhstan* OR Kazak OR Kenya* OR Kenian* OR Kiribati* OR “i-kiribati” OR Korea* OR Kosovo* OR Kosovar* OR Kyrgyz OR Kyrgyzstan OR Kirghiz* OR Kirgiz* OR Laos OR Laotian* OR Lebanon OR Lebanese* OR Lesotho OR Basutoland OR Mosotho* OR Basotho OR Basuto OR Sotho* OR “Liberia Sudan” OR Libya OR Libyan* OR Madagascar OR “Malagasy Republic” OR Madagascan* OR Malagasy OR Malawi* OR Malaysia* OR Malaya OR “North Borneo” OR Maldives OR Maldivian OR Dhivehin OR Mali OR Malian* OR “Marshall Islands” OR Marshallese* OR Mauritania* OR mauretania OR Micronesia* OR Mongolia* OR Morocco OR Moroccan* OR Mozambique OR mocambique OR Mozambican* OR Myanmar OR Burma OR Burmese* OR Myanmarese* OR Myanmese* OR Namibia* OR “southwest africa” OR Nepal* OR Nicaragua* OR Niger* OR Nigeria* OR Macedonia* OR “former yugoslav republic” OR Pakistan* OR Panama* OR “Papua New Guinea*” OR Paraguay* OR Peru* OR Philippine* OR Filipin* OR Romania* OR Rumania* OR “Russian Federation” OR Russia* OR Siberia OR Rwanda* OR Samoa* OR “Sao Tome*” OR Santomean* OR Senegal* OR Serbia* OR “Sierra Leone*” OR “Solomon Island*” OR Somali* OR “South Africa*” OR “South Sudan*” OR “Sri Lanka*” OR ceylon OR “Saint Lucia*” OR “St. Lucia*” OR “Saint Vincent and the Grenadines” OR Vincentians OR Suriname* OR Surinam OR “Syrian Arab Republic” OR Syrian* OR Tajikistan OR Tadzhik OR Tadzhikistan OR Tajik* OR Tanzania OR Tanganyika OR Zanzibar OR Tanzanian* OR Thailand OR siam OR Thai OR Thais OR “Timor-Leste” OR Timor OR Timorese* OR Togo OR Togolese* OR Tonga OR Tongan* OR Batonga* OR Tunisia OR tunesia OR Tunisian* OR Turkey OR Turk* OR Istanbul OR Uganda* OR Ukraine OR Ukraina OR Ukrainian* OR Uzbekistan OR Uzbek* OR Vanuatu* OR “Ni-Vanuatu” OR “Ni-Van” OR Vietnam OR “Viet Nam” OR Vietnamese* OR “West Bank” OR Gaza OR Yemen OR Aden OR Yemeni* OR Zambia* OR “northern rhodesia” OR Zimbabwe OR Rhodesia OR Zimbabwean* OR “Dominican Republic” OR Dominican* OR Mauritius OR Mauritian* OR Turkmenistan OR Turkmen* OR Turkmenian* OR “Equatorial Guinea” OR Mexico OR Mexican* OR Tuvalu OR Tuvaluan* OR Ecuador* OR Moldova OR Moldavia OR Moldavian* OR Fiji* OR Montenegro OR Montenegrin*) OR TI (“Sao Tome” N1 Principe) OR AB (Afghanistan OR Afghan OR Afghans OR Albania* OR Algeria* OR “American Samoa*” OR Angola* OR Argentina OR Argentinian* OR Armenia* OR Azerbaijan* OR Bangladesh* OR Belarus* OR Belize* OR Benin* OR Bhutan* OR Bolivia* OR “Bosnia and Herzegovina” OR Bosnian* OR Bosniak* OR Botswana OR Bechuanaland OR Brazil OR Brazilian* OR Bulgaria* OR “Burkina Faso” OR “upper volta” OR Burundi OR “Cabo Verde” OR “Cape Verde” OR Cambodia OR kampudja OR “khmer republic” OR Cambodian* OR Cameroon* OR cameroun* OR “Central African Republic” OR “Centrafrican Republic” OR Centrafrique OR “Central African Empire” OR “Central African*” OR Chad OR Tchad OR Chadian* OR China OR Beijing OR Chinese* OR Colombia* OR Columbia* OR Comoros OR “comoro islands” OR Congo OR Congolese* OR Costa Rica* OR “Ivory Coast” OR Ivorian* OR Cuba* OR Djibouti* OR Dominica* OR Egypt* OR “united arab republic” OR “El Salvador” OR Salvador* OR Eritrea* OR Eswatini OR Swaziland OR Swazi* OR Ethiopia* OR Gabon* OR Gaboon OR Gambia* OR “Georgia republic” OR Georgian* OR Ghana* OR Grenada OR Grenadian* OR Guatemala* OR Guinea* OR Guinea-Bissau OR Guyana OR Guyanese OR Haiti* OR Honduras OR Honduran* OR India OR “Indian Union” OR Indonesia* OR Iran OR Persia* OR Iranian* OR Iraq* OR Jamaica* OR Jordan* OR Kazakhstan* OR Kazak OR Kenya* OR Kenian* OR Kiribati* OR “i-kiribati” OR Korea* OR Kosovo* OR Kosovar* OR Kyrgyz OR Kyrgyzstan OR Kirghiz* OR Kirgiz* OR Laos OR Laotian* OR Lebanon OR Lebanese* OR Lesotho OR Basutoland OR Mosotho* OR Basotho OR Basuto OR Sotho* OR “Liberia Sudan” OR Libya OR Libyan* OR Madagascar OR “Malagasy Republic” OR Madagascan* OR Malagasy OR Malawi* OR Malaysia* OR Malaya OR “North Borneo” OR Maldives OR Maldivian OR Dhivehin OR Mali OR Malian* OR “Marshall Islands” OR Marshallese* OR Mauritania* OR mauretania OR Micronesia* OR Mongolia* OR Morocco OR Moroccan* OR Mozambique OR mocambique OR Mozambican* OR Myanmar OR Burma OR Burmese* OR Myanmarese* OR Myanmese* OR Namibia* OR “southwest africa” OR Nepal* OR Nicaragua* OR Niger* OR Nigeria* OR Macedonia* OR “former yugoslav republic” OR Pakistan* OR Panama* OR “Papua New Guinea*” OR Paraguay* OR Peru* OR Philippine* OR Filipin* OR Romania* OR Rumania* OR “Russian Federation” OR Russia* OR Siberia OR Rwanda* OR Samoa* OR “Sao Tome*” OR Santomean* OR Senegal* OR Serbia* OR “Sierra Leone*” OR “Solomon Island*” OR Somali* OR “South Africa*” OR “South Sudan*” OR “Sri Lanka*” OR ceylon OR “Saint Lucia*” OR “St. Lucia*” OR “Saint Vincent and the Grenadines” OR Vincentians OR Suriname* OR Surinam OR “Syrian Arab Republic” OR Syrian* OR Tajikistan OR Tadzhik OR Tadzhikistan OR Tajik* OR Tanzania OR Tanganyika OR Zanzibar OR Tanzanian* OR Thailand OR siam OR Thai OR Thais OR “Timor-Leste” OR Timor OR Timorese* OR Togo OR Togolese* OR Tonga OR Tongan* OR Batonga* OR Tunisia OR tunesia OR Tunisian* OR Turkey OR Turk* OR Istanbul OR Uganda* OR Ukraine OR Ukraina OR Ukrainian* OR Uzbekistan OR Uzbek* OR Vanuatu* OR “Ni-Vanuatu” OR “Ni-Van” OR Vietnam OR “Viet Nam” OR Vietnamese* OR “West Bank” OR Gaza OR Yemen OR Aden OR Yemeni* OR Zambia* OR “northern rhodesia” OR Zimbabwe OR Rhodesia OR Zimbabwean* OR “Dominican Republic” OR Dominican* OR Mauritius OR Mauritian* OR Turkmenistan OR Turkmen* OR Turkmenian* OR “Equatorial Guinea” OR Mexico OR Mexican* OR Tuvalu OR Tuvaluan* OR Ecuador* OR Moldova OR Moldavia OR Moldavian* OR Fiji* OR Montenegro OR Montenegrin*) OR AB (“Sao Tome” N1 Principe))  AND  (MH "Discrimination") OR (MH "Communication") OR (MH "Mentorship") OR (MH "Patient Centered Care") OR (MH "Quality Management, Organizational") OR (MH "Root Cause Analysis") OR (MH "Social Responsibility") OR (MH "Problem-Based Learning") OR TI ((Childcentered OR “child centered care” OR clientcentered OR “client centered” OR responsive) N3 (care OR “health service*” OR healthservice*)) OR TI ((“in service” OR inservice OR “in-service” OR rights) N3 training) OR TI ((judgmental OR discriminatory) N2 attitude*) OR TI ((quality OR QI) N3 (improvement OR management OR approach)) OR TI (social N3 (accountability OR responsibility* OR obligation*)) OR TI (“Community Score Card” OR discrimination OR “enhanced patient care” OR communication OR disclosure OR “teach-back” OR KidzAlive OR “KidzAlive-trained” OR mentor* OR “patient engagement” OR “patient-centered” OR “patient centered” OR “Patient-Centred” OR “patient centered” OR “patient centred” OR “person centered” OR “person centred” OR “person-centered” OR “person-centred” OR “provider training” OR “root cause analysis” OR “root cause analyses” OR “sensitisation training*” OR “sensitivity training*” OR “Team-based learning” OR “team based learning” OR “tests of change”) OR AB ((Childcentered OR “child centered care” OR clientcentered OR “client centered” OR responsive) N3 (care OR “health service*” OR healthservice*)) OR AB ((“in service” OR inservice OR “in-service” OR rights) N3 training) OR AB ((judgmental OR discriminatory) N2 attitude*) OR AB ((quality OR QI) N3 (improvement OR management OR approach)) OR AB (social N3 (accountability OR responsibility* OR obligation*)) OR AB (“Community Score Card” OR discrimination OR “enhanced patient care” OR communication OR disclosure OR “teach-back” OR KidzAlive OR “KidzAlive-trained” OR mentor* OR “patient engagement” OR “patient-centered” OR “patient centered” OR “provider training” OR “root cause analysis” OR “root cause analyses” OR “sensitisation training*” OR “sensitivity training*” OR “Team-based learning” OR “team based learning” OR “tests of change”)  AND  ((MH "Outpatient Service") OR (MH "Attitude of Health Personnel") OR (MH "Clinical Nurse Specialists") OR (MH "Novice Clinicians") OR (MH "Expert Clinicians") OR (MH "Physician-Patient Relations") OR (MH "Professional-Patient Relations") OR (MH "Nurse-Patient Relations") OR (MH "Health Care Delivery") OR (MH "Health Facilities") OR (MH "Health Personnel") OR (MH "Health Services") OR (MH "Patient Care") OR (MH "Primary Health Care") OR TI ((“ambulatory care” OR ambulant OR outpatient OR “out-patient” OR “out patient”) N3 (facility* OR “operation room” OR care OR department* OR clinic* OR unit*)) OR TI (day N2 (clinic OR clinics OR hospital*)) OR TI (policlinic OR polyclinic OR surgicenters OR “clinical care” OR clinician* OR “patient provider*”) OR TI (attitude N3 (“health personnel” OR “health care personnel” OR “healthcare personnel”)) OR TI ((“clinician-patient” OR “doctor patient” OR “patient doctor” OR “hospital patient” OR “patient physician” OR “physician patient” OR “physician-patient” OR “patient staff” OR “patient therapist” OR therapeutic OR “therapist patient” OR “professional-patient” OR “patient-health” OR “patient-healthcare” OR “nurse patient” OR “nurse-patient” OR “patient-nurse”) N3 (relation* OR contact)) OR TI (“bedside psychology”) OR TI ((health OR healthcare) N3 (delivery OR facility*)) OR TI ((health OR healthcare) N2 (personnel OR practitioner* OR professional* OR provider* OR worker* OR aid*)) OR TI ((health OR healthcare OR physician) N3 (center* OR centre* OR clinic OR clinics OR institute* OR service* OR agenc* OR setting*)) OR TI ((patient OR continuity OR episode* OR “patient-centered”) N3 (care OR management)) OR TI ((primary OR “first line”) N3 (care OR caregivers OR healthcare)) OR TI (provider N2 (attitude* OR service*)) OR AB ((“ambulatory care” OR ambulant OR outpatient OR “out-patient” OR “out patient”) N3 (facility* OR “operation room” OR care OR department* OR clinic* OR unit*)) OR AB (day N2 (clinic OR clinics OR hospital*)) OR AB (policlinic OR polyclinic OR surgicenters OR “clinical care” OR clinician* OR “patient provider*”) OR AB (attitude N3 (“health personnel” OR “health care personnel” OR “healthcare personnel”)) OR AB ((“clinician-patient” OR “doctor patient” OR “patient doctor” OR “hospital patient” OR “patient physician” OR “physician patient” OR “physician-patient” OR “patient staff” OR “patient therapist” OR therapeutic OR “therapist patient” OR “professional-patient” OR “patient-health” OR “patient-healthcare” OR “nurse patient” OR “nurse-patient” OR “patient-nurse”) N3 (relation* OR contact)) OR AB (“bedside psychology”) OR AB ((health OR healthcare) N3 (delivery OR facility*)) OR AB ((health OR healthcare) N2 (personnel OR practitioner* OR professional* OR provider* OR worker* OR aid*)) OR AB ((health OR healthcare OR physician) N3 (center* OR centre* OR clinic OR clinics OR institute* OR service* OR agency* OR setting*)) OR AB ((patient OR continuity OR episode* OR “patient-centered”) N3 (care OR management)) OR AB ((primary OR “first line”) N3 (care OR caregivers OR healthcare)) OR AB (provider N2 (attitude* OR service*))))  AND  (MH "Patients") OR (MH "Men Who Have Sex With Men") OR (MH "Sexual and Gender Minorities") OR (MH "Social Workers") OR (MH "Sex Work") OR (MH "LGBTQ+ Persons") OR TI (men N3 “sex with men”) OR TI (call girl* OR prostitute* OR “sex work*” OR prostitution) OR TI ((HIV OR HIV+ OR “Human immunodeficiency virus”) N3 (child OR children OR adolescent* OR teen OR teenager OR youth* OR pregnancy OR pregnant OR breastfeed* OR “breast feed*”)) OR TI (patient*) OR TI (LGBTQIA+ OR Bisexual* OR gay* OR lesbian* OR pansexual* OR queer* OR asexual* OR transgender* OR intersex* OR “gender-expansive” OR questioning OR GLBTI* OR GLBTQ* OR trans OR genderqueer OR “two-spirit*” OR “two spirit*” “2 spirit*” OR “2-spirit*” OR transsexual OR “gender diverse” OR LGBTI* OR LGBTQ* OR LGBTT*) OR AB (men N3 “sex with men”) OR AB (call girl* OR prostitute* OR “sex work*” OR prostitution) OR AB ((HIV OR HIV+ OR “Human immunodeficiency virus”) N3 (child OR children OR adolescent* OR teen OR teenager OR youth* OR pregnancy OR pregnant OR breastfeed* OR “breast feed*”)) OR AB (patient*) OR AB (LGBTQIA+ OR Bisexual* OR gay* OR lesbian* OR pansexual* OR queer* OR asexual* OR transgender* OR intersex* OR “gender-expansive” OR questioning OR GLBTI* OR GLBTQ* OR trans OR genderqueer OR “two-spirit*” OR “two spirit*” “2 spirit*” OR “2-spirit*” OR transsexual OR “gender diverse” OR LGBTI* OR LGBTQ* OR LGBTT*) |
| 10.1.23 | ClinicalTrials.gov | (HIV OR antiretroviral) AND (“low income country*” OR “middle income country*”) AND (“Patient provider*” OR “health care worker*” OR “health care facility*”) AND (“provider training” OR attitude* OR intervention*) AND (LGBTQIA OR “sex worker*”) |

## Table S2. Dimensions of the Integrative Model of Patient-Centeredness by Scholl et al. (2014)

| **Dimension** | **Brief description** |
| --- | --- |
| **Principles** | |
| Essential characteristics of the clinician | A set of attitudes towards the patient (e.g., empathy, respect, honesty) and oneself (self-reflectiveness) as well as medical competency |
| Clinician-patient relationship | A partnership with the patient that is characterized by trust and caring |
| Patient as a unique person | Recognition of each patient's uniqueness (individual needs, preferences, values, feelings, beliefs, concerns and ideas, and expectations) |
| Biopsychosocial perspective | Recognition of the patient as a whole person in his or her biological, psychological, and social context |
| **Enablers** | |
| Clinician-patient communication | A set of verbal and nonverbal communication skills |
| Integration of medical and non-medical care | Recognition and integration of non-medical aspects of care (e.g., patient support services) into health care services |
| Teamwork and teambuilding | Recognition of the importance of effective teams characterized by a set of qualities (e.g. respect, trust, shared responsibilities, values, and visions) and facilitation of the development of such teams |
| Access to care | Facilitation of timely access to healthcare that is tailored to the patient |
| Coordination and continuity of care | Facilitation of healthcare that is well coordinated (e.g. regarding follow-up arrangements) and allows continuity |
| **Activities** | |
| Patient information | Provision of tailored information while considering the patient's information needs and preferences |
| Patient involvement in care | Active involvement of and collaboration with the patient regarding decisions related to the patient's health while considering the patient's preference for involvement |
| Involvement of family- friends | Active involvement of and support for the patient's relatives and friends to the degree that the patient prefers |
| Patient empowerment | Recognition and active support of the patient's ability and responsibility to self-manage his or her disease |
| Physical support | A set of behaviours that ensures physical support for the patient (e.g. pain management, assistance with daily living needs) |
| Emotional support | Recognition of the patient's emotional state and a set of behaviour that ensures emotional support for the patient |

## Table S3: HIV care continuum and PCC outcomes of included studies by outcomes

| **Study** | **Outcome (definition)** | **Follow up time (months)** | **Intervention group**  n/N (%) | **Comparison group**  n/N (%) | **Effect size** (**95% CI)** | **P value** | **Key findings** |
| --- | --- | --- | --- | --- | --- | --- | --- |
| **Linkage to HIV care** | | | | | |  |  |
| Ayieko 2019 (SEARCH) | Linkage to HIV care (first HIV clinic visit after the community-based test) | 12 | 1503/2051 (73.3) | NA | NA | NA | Nearly ¾ of individuals were linked to care (overall) |
| Ayieko 2019 (SEARCH) | Linkage to HIV care (linked to care within 7 days of HIV diagnosis) | 7 days | 1019/2051 (49.7) | NA | NA | NA | 50% were linked to care within one week |
| Kerrigan 2019 | Linkage to HIV care (ever linked to HIV care) | 18 | 72/91 (79.1) | 44/80 (55.0) | RR 1.44 (CI: ND) | 0.002 | The intervention had a significant positive effect |
| Ruria 2017 | Linkage to HIV care (completed first appointment with an HIV care provider following a positive HIV test) | 18 | 544/559 (97.3) | 222/393 (56.5) | ND | < 0.0001 | The intervention had a significant positive effect |
| Smith 2019† | Linkage to HIV care (ND) | 8 | 98% post | 63% pre | ND | ND | 98% linkage to care in intervention, 63% in pre-intervention |
| **ART initiation** | | | | | |  |  |
| Pascoe 2019 | ART initiation (initiated on ART within 30 days of eligibility) | 12 | 298/360 (83.0) | 303/368 (82.0) | aRD 6.3% (-0.6-13.3) | ND | No significant difference between groups. |
| Penfold 2019 | ART initiation (ND) | 48 | 509/ND post | 69/ ND (pre) | ND | ND | The absolute number of individuals who were initiated on ART increased over time (no information on denominator provided) |
| Ruria 2017 | ART initiation within 1 month | 18 | 430/544 (79.0) | 160/222 (72.1) | ND | P>0.05 | No significant difference between groups |
| **Adherence to ART** | | | | | |  |  |
| Erb 2017† | ART adherence (self-reported ≥ 1 dose missed in the last 4 weeks) | 6-9 | 16/280 (5.7) | 10/299 (3.3) | ND | 0.2 | No significant difference between groups. |
| Jones 2013† | ART adherence (self-reported no missed doses in the previous 3 months) | 6 | 52/77 (67.5) [group intervention] | 48/83 (57.8) [Individual intervention] | ND | 0.91 | No significant difference between groups. |
| Jones 2016† | ART adherence (0-100 rating of percent adherence in the last 4 weeks) | 9 | Pt + HCW active: Mean (SD): 82.0 (5.6) n=30 | Pt active/HCW inactive: Mean (SD): 63.0 (5.6) n=31 | ND | 0.47 | No significant difference between groups. |
| Kerrigan 2019 | ART adherence (within the last 4 days) | 18 | 65/91 (71.4) | 37/80 (46.2) | RR 1.54 | <0.01 | The intervention had a significant positive effect |
| Onokala 2015 | ART adherence (missed ≤ 3 doses per month) | 48 | 1195/2267 (88.0) | 907/2267 (40.0) | ND | ND | 88% ART initiation in intervention, 40% in control |
| Puttkammer 2020 | ART adherence (high adherence, “proportion of days covered” ≥ 90%) | 36 | 40/362 (11.0) pre  25/81 (30.9) post | 71/366 (19.4) pre  11/65 (16.9) post | aIRR 4.00 (1.91- 8.38) | <0.001 | The intervention had a significant positive effect |
| Puttkammer 2020 | ART adherence (high adherence, never >7 days late for ART pickup) | 36 | 116/362 (32.0) pre  42/81 (51.9) post | 165/366 (45.0) pre  24/65 (36.9) post | aIRR 2.16 (1.42-3.28) | <0.001 | The intervention had a significant positive effect |
| **Retention in Care** |  |  |  |  |  |  |  |
| Abboah-Offei† 2020 | Retention into the study | 3 | 30/30 (100.0) | 28/30 (93.3) | ND | ND | Retention was high (>90%) across both groups. |
| Hickey 2020‡ (SEARCH) | Time in care defined as a proportion of follow-up time that patients were in care (ART experience with baseline viremia) | 36 | 27/330 (81.8) | 173/238 (72.7) | RR 1.11 (1.02-1.19) | ND | The intervention had a significant positive effect |
| Hickey 2020‡ (SEARCH) | Time in care- as a proportion of follow-up time that patients were in care (ART naïve with baseline CD4<350) | 36 | 380/514 (74.0) | 235/351 (67.0) | RR 1.10 (1.03-1.17) | ND | The intervention had a significant positive effect |
| Hickey 2020‡ (SEARCH) | Time in care defined as a proportion of follow-up time that patients were in care (ART experienced with baseline VS) | 36 | 1416/1646 (86.0) | 1063/1312 (81.0) | RR 1.07 (1.01-1.13) | ND | The intervention had a significant positive effect |
| Kays 2021 | Retention in PMTCT services (attended the most recent scheduled visit within 6 months) | 6 | 431/606 (71.1) | 352/627 (56.1) | ND | 0.53 | No difference between groups. |
| Keene 2019† | Retention in care (not defined) | ND | 171/196 (87.2) | NA | NA | NA | 87% retention |
| Kerrigan 2019 | Retention in care (In care during last 6 months) | 18 | 70/91 (76.9) | 41/80 (51.2) | RR 1.5 (CI: ND) | <0.001 | The intervention had a significant positive effect |
| Moucheraud 2020 | Appointment attendance in Uganda (attended appointment on scheduled day) | 18-24 | ND/1650 | ND/51 | aOR 1.62 (0.37-7.02) | ND | No significant difference between groups. |
| Moucheraud 2020 | Appointment attendance in Tanzania (attended appointment on scheduled day) | 18-24 | ND/3416 | ND/119 | aOR 3.53 (2.15-5.77) | <0.001 | The intervention had a significant positive effect |
| Pascoe 2019 | Retention in Care (Not transferred, lost-to-follow-up, failure to attend ART visit, or died) | 12 | 240/360 (66.7) | 254/367 (69.2) | aRD -3.6% (-11.1-3.9) | ND | No significant difference between groups. |
| Reif 2019 | Retention in care (clinic visit between 11 and 13 months from HIV testing) | 12 | 43/50 (86.0) | NA | NA | NA | 86% retention |
| Ruria 2017 | Retention in care (being in care with a record of having been dispensed ART at the time of the evaluation) | 6 | 144/146 (98.6) | 117/215 (54.4) | ND | <0.0001 | The intervention had a significant positive effect |
| Teasdale 2019† | Retention in care (retained in antenatal care within one-month window before and after retention endpoint) | 6 | 42/56 (75.0) | 29/41 (70.7) | ND | 0.54 | No significant difference between groups. |
| Wachira 2022 i† | Retention in care (2 consecutive visits within 7 days of scheduled clinic appointment date) | 6 | 108/110 (98.2) | 101/108 (93.3) | aOR 2.70 (0.56-20.00) (inverse OR) | ND | No significant difference between groups. |
| Wachira 2022 ii† | Retention in care (2 consecutive visits within 7 days of scheduled clinic appointment date) | 6 | 105/110 (95.1) | 101/108 (93.3) | ND | ND | No significant difference between groups. |
| Zanoni 2017 | Retention in care (one clinic visit or pharmacy refill) | 67 | 84/88 (95.5) | 130/153 (85.0) | OR 3.7 (1.2-11.1) | 0.018 | The intervention had a significant positive effect |
| **Viral load coverage** | | | | | | | |
| Emerenini 2021 | Viral load coverage (not defined) | 6 | 3340/4294 (77.8) (all age groups) | 977/3298(29.6) | ND | <0.001 | The intervention had a significant positive effect |
| **Viral Suppression** | | | | | |  |  |
| Balzer 2017 (SEARCH) | Viral suppression (<500 copies/mL) | 24 | 412/665 (62.0) | 233/665 (35.0) | ND | ND | SEARCH increased overall HIV viral suppression among HIV+ children from 35% to 62%. |
| Emerenini 2021 | Viral suppression (not defined) | 6 | 3341/3977 (84.0) | 1831/2828 (64.8) | ND | 0.03 | The intervention had a significant positive effect |
| Erb 2017† | Virological suppression (<1000 copies/mL) | 6-9 | 258/284 (90.8) | 274/297 (92.3) | OR: 0.92 (NR) | 0.5 | No difference between groups. |
| Hickey 2020 (SEARCH) | Viral suppression (<500 copies/mL) | 36 | 2373/2644 (90.0) | 1970/2264 (87.0) | RR 1.03 (1.01-1.06) | ND | The intervention had a significant positive effect |
| Jones 2016† | Viral suppression (not defined) | 9 | Pt+HCW active  14/30(46.7) | Pt active/HCW  15/31 (48) | ND | 0.996 | No significant difference between groups. |
| Keene 2019† | Viral suppression (<1000 copies/mL) | 3 | 36/46 (78.3) | NA | NA | NA | 78% viral suppression |
| Kerrigan 2019 | Viral suppression (<400 copies/mL) | 18 | 46/91 (50.6) | 36/80 (47.4) | RR 1.05 (ND) | 0.74 | No significant difference between groups. |
| Mburu 2019† | Viral suppression (<1000 copies/mL) | ND | 958/1345 (71.2) | 1435/2195 (65.4) | aOR 0.97 (0.72-1.30) | 0.84 | No significant difference between groups. |
| Mwangwa 2022 | Viral Suppression (<400 copies/ml) | 24 | 890/915 (87.9) | 734/918 (80.0) | aRR 1.10 (1.03-1.16) | 0.002 | The intervention had a significant positive effect |
| Pascoe 2019 | Viral suppression (<400 copies/mL) | 12 | 221/362 (61.1) | 235/368 (63.9) | aRD -1.9% (-9.1-5.4) | ND | No significant difference between groups. |
| PATA 2017 | Viral Suppression (not defined) | 12 | ND/ND (88.4) post | ND/ND (80.0) pre | ND | ND | An increase in viral suppression from 80% to 88.4%. |
| Puttkammer 2020 | Viral Suppression (<1000 copies/mL) | 36 | 37/43 (86.0) pre  36/45 (80.0) post | 125/143 (87.4) pre  43/56 (76.8) post | aIRR 1.15 (0.92-1.45) | 0.21 | No significant difference between groups. |
| Reif 2019 | Viral Suppression (<1000 copies/mL) | 12 | 13/40 (32.5) | NA | NA | NA | 32.5% viral suppression |
| Wachira 2022 i† | Viral Suppression (< 400 copies/mL) | 6 | 93/110 (84.4) | 80/108 (64.4) | aOR 2.78 (1.39-5.56) (inverse OR) | <0.05 | The intervention had a significant positive effect |
| Wachira 2022 ii† | Viral Suppression (<400 copies/mL) | 6 | 92/110 (83.7) | 80/108 (64.4) | ND | <0.05 | The intervention had a significant positive effect |
| Zanoni 2017 | Viral Suppression (<400 copies/mL) | 6 | 80/88 (90.0) | 116/153 (75.8) | OR 2.5 (1.1-5.8) | 0.028 | The intervention had a significant positive effect |
| **PCC Outcome- Patient-provider communication** | | | | | |  |  |
| Abboah-Offei† 2020 | Consultation and relational empathy (CAREM). Standardized ax | 3 | Mean (SD): 33.0 (1.4) N=28 | Mean (SD): 7.0 (1.6) N= 30 | MD 1.0 (0.45–1.55) | <0.001 | The intervention had a significant positive effect |
| Laterra 2020  (CARE CSC) | Patient-provider communication (disclosure support and maintenance of confidentiality of HIV status). Not standardized ax | 12 | 69 points post | 42 points pre | 64% increase | <0.05 | The intervention had a significant positive effect |
| MacLachlan† 2016a | Patient/Provider Interactions (Roter Interaction Analysis System (RIAS) code to quantify patient-provider conversations) - All patient question asking. Standardized ax | 6 | Mean (SD): 2.10 (1.53) N=160 | Mean (SD): 1.05 (1.44) N=129 | aD: 0.48 (0.11-0.85) | 0.012 | The intervention had a significant positive effect |
| Jones 2016† | Provider actions related to the treatment. Information not provided on measurement. | 9 | Mean (SD): 11.3 (0.5) N=60 | Mean (SD): 9.5 (0.6) N=60 | ND | 0.02 | The intervention had a significant positive effect |
| **PCC Outcome- Patient satisfaction** | | | | | |  |  |
| Abboah-Offei† 2020 | Patient experience questionnaire (PEQ)  Higher score=better outcomes. Standardized ax | 3 | Mean (SD):  24.0 (2.4) N=28 | Mean (SD): 32.0 (1.5) N= 30 | MD 0.8 (0.27-1.31) | <0.001 | The intervention had a significant positive effect |
| Jones 2016† | Satisfaction with provider relationship. Information not provided on measurement. | 9 | Mean (SD): 14.2 (0.4) N= 60 | Mean (SD): 12.6 (0.5) N= 60 | ND | 0.01 | The intervention had a significant positive effect |
| Galy 2018 | Satisfaction with the intervention. Not standardized ax | ND | ND/139 | NA | NA | NA | 88.8% of participants were satisfied with the intervention |
| **PCC Outcome- Perceived Quality of Care** | | | | | |  |  |
| Laterra 2020  (CARE CSC) | Quality and Professionalism of the provider (attitude and commitment of PMTCT service providers). Not standardized ax | 12 | 85 points post | 58 points pre | 47% increase | <0.05 | The intervention had a significant positive effect |
| Laterra 2020  (CARE CSC) | Quality and Professionalism of the provider (prevalence of stigma and discriminatory behaviours towards women living with HIV). Not standardized ax | 12 | 80 points post | 49 points pre | 63% reduction | <0.05 | The intervention had a significant positive effect |
| Laterra 2020  (CARE CSC) | Perceived quality of care (Convenient and timely access to services). Not standardized ax | 12 | 86 points post | 64 points pre | 34% increase | <0.05 | The intervention had a significant positive effect |
| **PCC Outcome- Other** | | | | | |  |  |
| Abboah-Offei† 2020 | Person-centered measures (PO)  Higher scores = better outcomes. Standardized ax | 3 | Mean (SD): 30.0 (3.9) N=28 | Mean (SD): 16.0 (3.8) N=30 | MD 0.7 (0.17-1.23) | <0.001 | The intervention had a significant positive effect |
| Abboah-Offei† 2020 | Health-related quality of life (MOS-HIV scale) Higher scores = better outcomes. Standardized ax | 3 | Mean (SD): 83.0 (2.9) N=28 | Mean (SD): 53.2 (3.9) N=30 | MD 0.7 (0.17-1.23) | <0.001 | The intervention had a significant positive effect |
| Abboah-Offei† 2020 | Physical, psychological symptoms, spiritual practical and emotional concerns, and psychosocial needs (APOS). Standardized ax  Lower scores = better outcomes | 3 | Mean (SD): 9 (1.7) N=28 | Mean (SD): 14 (1.7) N=30 | MD 0.7 (0.17-1.23) | <0.001 | The intervention had a significant positive effect |
| Jones 2016† | Self-efficacy (HIV treatment adherence self-efficacy scale). Standardized ax | 9 | Mean (SD): 105.2 (3.8) N=60 | Mean (SD): 99.8 (4.3) N=60 | ND | 0.31 | No significant difference between groups. |
| Jones 2016† | HIV-related knowledge (adapted from Personal HIV knowledge measure) | 9 | Mean (SD): 7.7 (0.2) N=60 | Mean (SD): 7.6 (0.2) N=60 | ND | 0.47 | No significant difference between groups. |
| Jones 2016† | Motivation for adherence (Life Windows Information Motivation Behavioral Skills ART Adherence Questionnaire). Standardized ax | 9 | Mean (SD): 35.4 (1.1) N=60 | Mean (SD): 36.0 (1.2) N=60 | ND | 0.26 | No significant difference between groups. |
| Jones 2016† | Symptoms of depression (Beck Depression inventory II). Standardized ax | 9 | Mean (SD): 3.3 (0.6) | Mean (SD):1.4 (0.6) | ND | P=0.03 | The intervention had a significant positive effect |
| Reif 2019 | Hopelessness defined as the proportion of adolescents feeling hopeless in the past 30 days. Information not provided on measurement. | 12 | 20% (9/45) | 38% (19/50) | ND | 0.05 | The intervention had a significant positive effect |
| Reif 2019 | Depression defined as the proportion of individuals who reported depression. Information not provided on measurement. | 12 | 17% (8/45) | 34% (17/50) | ND | 0.07 | No significant difference between groups. |
| Reif 2019 | Desire for emotional support defined as the proportion of adolescents reporting a desire for more emotional support from family and friends. Information not provided on measurement. | 12 | 50% (22/45) | 94% (47/50) | ND | <0.01 | The intervention had a significant positive effect |
| **Socio-Economic-related outcomes** | | | | | | | |
| Jakubowski 2022(SEARCH) | Probability of having spent money on healthcare between intervention and control communities in the previous 30 days | 36 | ND/1387 | ND/1409 | MD -12.7 (-22.4-0.6) | ND | Pts in intervention communities were 12.7% less likely to report spending money on healthcare. |
| Jakubowski 2022 (SEARCH) | Probability of sought health care for illness or injury between intervention and control communities in the previous 30 days | 36 | ND/1387 | ND/1409 | MD -10.3 (-22.0-0.1) | ND | Pts in intervention communities were 10.3% less likely to report seeking out health care for illness or injury |
| Jakubowski 2022 (SEARCH) | Probability of lost time from activities due to illness between intervention and control communities in the previous 30 days. | 36 | ND/1387 | ND/1409 | MD -7.1 (-17.7-0.7) | ND | Pts in intervention communities were 7.1% less likely to report loss of time from activities. |
| Jakubowski 2022 (SEARCH) | Probability of increased employment (any work) between intervention and control communities in the previous 7 days. | 36 | ND/1387 | ND/1409 | MD 9.7 (2.1-18.3) | ND | Pts in the intervention communities were 9.7% more likely to be employed. |

CI, confidence interval; OR, odds ratio, RR, risk ratio, HR, hazard ratio; MD, mean difference; IRR, incidence rate ratio; RD, risk difference; aOR, adjusted OR; aRR, adjusted risk ratio; aRD, adjusted risk difference; aDRD, adjusted difference in risk differences; aD, adjusted difference; SD, standard deviation; SE, standard error; NA, Not applicable; ND, Not described; n, number of events; N, number of participants; ART, antiretroviral therapy; Pt, patient; HCW, health care worker/provider.

†PCC is the main intervention component.

‡Data provided for each group but not overall

## Table S4: Patient-Centered Care Intervention Components aimed at Patient-Provider Relationship

| **Study Name** | **Actor** | **Actions- PCC Intervention** | **Dose** | **Location** | **Mechanism of PCC pt-provider interactions** | **Overall findings** |
| --- | --- | --- | --- | --- | --- | --- |
| 1.Abboah-Offei 2020† | HCP | HCP received 3 session training program on PCC and communication and participants receive clinical care from the trained HCP. | Baseline + repeated | Community | Those in the intervention arm received care from HCP who received 3 trainings on PCC to improve outcomes for PLWH. | Community person-centred intervention is feasible and acceptable to PLWH and HCP. |
| 2.Ayieko 2019 (SEARCH) | Clinic Staff | Streamlined care intervention in community clinics (PCC, efficient visits, clinic access, reminders, and counselling). | Baseline | Community | Training providers and structural changes in clinic to improve friendliness of care and Pt-centeredness of viral load counselling. | Using a multicomponent linkage strategy, high proportions of PLWH but not in care were linked rapidly after HIV testing. |
| 3.Balzer 2017 (SEARCH) | Clinic staff | Streamlined care intervention in community clinics (PCC, efficient visits, clinic access, reminders, and counselling). | Baseline + repeated | Community | Training providers and structural changes in clinic to improve friendliness of care and Pt-centeredness of viral load counselling. | SEARCH increased overall HIV viral suppression among HIV+ children from 35% to 62%. |
| 4.Emerenini 2021 | Clinic Staff | Provide adolescent-friendly services. | ND | Facility | Provided adolescent-friendly services to eliminate barriers and develop strong relationships with clinical staff. | OTZ was associated with a significant improvement in VL testing coverage and viral suppression. |
| 5.Erb 2017† | HCP | Provider education and training on Pt-centered communication and the provision of an adherence assessment checklist. | Baseline | Facility | Supportive provider-pt communication at every pt-provider interaction to improve the ability of HCPs to elicit Pt’s reports of problems with adherence. | There was no difference between groups for adherence and viral suppression. |
| 6.Galy 2018 | HCP | Consultations on knowledge and skills related to HIV and treatment management, deciding on educational goals, use of tools to educate, and then evaluation of educational objectives. | Baseline + repeated | Facility | Educating and bringing awareness to Pts of HIV-related issues in order to build capacity to manage HIV disease and increase demand for care. | Practical skills like active listening, know-how and a space for discussion appear more important for Pts than knowledge on disease or treatments. |
| 7.Hickey 2020  (SEARCH) | ND | Streamlined care intervention in community clinics (PCC, efficient visits, clinic access, reminders, and counselling). | ND | Community | Training providers and structural changes in clinic to improve friendliness of care and Pt-centeredness of viral load counselling | Streamlined care was associated with more time in care across the different groups and higher overall suppression. |
| 8.Jakubowski 2022 (SEARCH) | Clinic Staff | Streamlined care intervention in community clinics (PCC, efficient visits, clinic access, reminders, and counselling). | Baseline + repeated | Community | Training providers and structural changes in clinic to improve friendliness of care and Pt-centeredness of viral load counselling. | Found significant improvements in economic status after 3 years for HIV-positive individuals living in intervention communities compared with those in control communities. |
| 9..Jones 2013† | Patients | Pts trained in health literacy and medication adherence, effective communication with providers through training sessions. | Baseline + repeated | Facility | Improved Pt-provider communication skills; mutual exchange of thoughts, attitudes, and feelings regarding adherence, service delivery, and access to care | Irrespective of the treatment group (individual or group intervention), there no difference in adherence at the end of the f/up. |
| 10..Jones 2016† | Clinic Staff | Train HCWs in motivational interviewing (stimulate Pt motivation and promote behaviour change) | Baseline + repeated | Facility | Improved provider attitude toward Pts and improved Pt-provider communication in consultation. | Pts reported greater satisfaction with their HCP and actions related to treatment compared to those in the control group. No difference between groups for ART adherence and viral suppression. |
| 11..Kays 2021 | HCP + Patients | Community Score Card created to bring Pts and service providers at the local level to collectively share feedback and improve the access and quality PMTCT of services | Baseline + repeated | Facility | Collectively share feedback on services to improve Pt experience. | The CSC approach could be a productive way to foster mutual trust and respect between HCPs and Pts, strengthen HCW-client relationships, & make meaningful improvements in service quality. No difference in retention between groups. |
| 12.Keene 2019† | HCP | Providers trained to welcome disengaged Pts back to care, normalize disengagement, support and empower them. | Baseline + repeated | Facility | Change provider attitude towards Pts who have disengaged from care in order to normalize disengagement and give support. | Welcome service Pts’ intervention had 87% retention and 78% viral suppression (no comparison group). |
| 13.Kerrigan 2019 | HCP | Providers were trained to administer health care to meet the unique needs of the FSW population through sensitivity training | Baseline + repeated | Community | Trained providers to address unique needs of FSWs to increase provider empathy, respect, compassion, & commitment to the Pt as well as highlight the social context influencing FSWs linkage to & retention in care. | The intervention had a significant positive effect on linkage, adherence and retention. There was no difference between groups for viral suppression. |
| 14.Laterra 2020 (CARE CSC) | HCP + Patients | Community Score Card created to bring Pts and service providers at the local level to collectively share feedback and improve the access and quality PMTCT of services. | Baseline + repeated | Facility | Collectively share feedback on services to improve Pt experience. | Community score card had a significant positive effect on PCC outcomes. |
| 15.Lowther 2018† | HCP | HIV clinic nurses received 2 weeks of palliative care training to enhance PCC. An experienced local hospice nurse provided the nurses with weekly supervision and mentoring. | Baseline + repeated | Facility | Trained nurses on palliative care met with Pts at 0 weeks, 2 weeks, 4 weeks and three subsequent monthly appointments in order in order to enhance Pt mental health. | The study enabled staff to identify and manage multidimensional problems and provide tailored health education and counselling. |
| 16.MacLachlan 2016† | Patients | Provide Pts with training on how to actively engage with providers, tools for improved Pt-provider communication, and how to overcome barriers to communication. | Baseline + repeated | Facility | Trained Pts in order to build skills for active care engagement and empowerment in their care. | The intervention had a significant positive affect on Pt’s asking providers questions. Increased engagement of Pts in clinical consultation can be achieved via a targeted training program. |
| 17.Mburu 2019† | HCP | Providers are trained on providing adolescent tailored health services including developmental, reproductive, sexual, psychosocial, and mental health as well as HIV prevention, care, and treatment. | Baseline | Facility | Trained providers to address the unique needs of adolescents and YA aims to increase provider empathy, communication, and clinical assessment of Pts to encourage adolescent retention in care. | There was no difference between groups for viral suppression. |
| 18.Medina-Marino 2020 | HCP | Nurses trained in providing support to Pts for disclosing their STI status to sexual partners as well as a two-week STI management and maternity care, including 10 hrs. of training in compassionate care and motivational interviewing skills. | Baseline + repeated | Facility | Nurses trained in compassionate care and motivational interviewing skills in order to support pregnant women in disclosing their STI status to sexual partners. | Supportive counselling by nurses at time of diagnosis helped to alleviate anxiety that women expressed toward disclosing their diagnoses, women felt more confident when approaching their treatment plan. |
| 19..Moucheraud 2020 | HCP | Training for providers on how to effectively provide self-management counselling. | ND | Facility | Creating a collaborative goal development plan between Pts and providers in order to increase Pt empowerment and use of shared decision-making. | There was a significant positive effect in appointment attendance in Tanzania. |
| 20.Mutambo 2020b | HCP | A combination of healthcare worker capacity building, the creation of child-friendly spaces, and child-friendly tools to address medical and social needs of children and adolescents. | Baseline + repeated | Facility | Training providers on the unique needs of children and adolescents and how to tailor services to this population to improve quality of care. | Child-friendly spaces promote children’s right to participation and agency in accessing care. |
| 21.Mwangwa 2022 | HCP | Pts in intervention clinics received life-stage specific assessment and counselling at the start of routine visits, choice of flexible clinic access HCPs had a secure mobile platform for inter-provider consultation. | Baseline + repeated | Facility | Recurrent re-evaluation of life stage events built relationships between AYAH and providers, which enabled them to promptly act when social structures change. | SEARCH Youth increased overall HIV viral suppression from 80% to 88%. |
| 22.Onokala 2015 | HCP + Patients | HCWs trained to implement integrated PCC model.  Pts and providers collectively set self-care plans and therapeutic goals. | ND | Facility | Improve Pt-provider interaction and Pt involvement. | ART Adherence initiation increased from 40% to 88% in the intervention. |
| 23.Pascoe 2019 | Clinic Staff | PCC ART counselling model, Pts supported to practice incorporating ART into their daily lives by addressing common barriers to starting ART and cognitive behavioural techniques. | Baseline + repeated | Facility | Worked with adherence counsellors to have shared decision making between Pt and provider in order to increase HIV outcomes. | There was no difference between groups for ART initiation, retention in care, and viral suppression. |
| 24.PATA 2017 | Clinic Staff | A combination of health provider sensitisation, dedicated adolescent-friendly service spaces, times, and location for improved adolescent and YA care. | Baseline + repeated | Facility | Have adolescent-friendly services and provider sensitisation to create a destigmatized environment intended to improve engagement in care. | There was an increase in viral suppression from 80% to 88.4% |
| 25.Penfold 2019 | HCP | Sensitivity training to prepare providers to work with FSWs | ND | Facility | Provided positive health care experiences in order to reduce stigma for FSWs. | There was a decrease in ART initiation post intervention from 15% to 9.6%. |
| 26.Puttkammer 2020 | HCP | An iSante EMR Alert for risk of treatment failure prompted HCPs to engage in a 5-step problem-solving counselling session with their Pts. | Baseline | Facility | Collaboratively developed goals between Pt and provider to emphasize the importance of adherence and Pt empowerment. | Intervention had a positive significant effect on ART adherence and no significant difference between groups for viral suppression. |
| 27.Reif 2019 | Clinic Staff | A monthly group visit consisting of 30 min of peer socialization and 30–45 min of peer-facilitated group counselling led by the FANMI nurse and social worker. | Baseline + repeated | Facility | Held monthly cohort meetings, integrated care, and provided services in a group setting to create long-term Pt-provider relationships, reduce isolation, inconvenience, and stigma. | This study is a promising model of adolescent HIV care that achieved high retention in care (86%) and improved mental health with a significant positive effect on hopelessness from 38% to 20%. |
| 28.Ruria 2017 | Program Staff | A school-based program that included peer-navigation, peer counselling & psychosocial support on HIV and SRH; creating a supportive environment to ensure ART adherence; supporting linkage to HCFs. | Baseline + repeated | Community | Provided adolescent-friendly services and peer support to create a supportive environment intended to improve engagement in care and decrease stigma. | The intervention had a positive significant effect on Linkage to HIV care (65.5% to 97.3%) and retention in care (54.4% to 98.6%). No significant difference in ART initiation. |
| 29.Smith 2019† | Clinic Staff | A roving clinical team provided onsite training to HCWs on adolescent friendly services, developing consultation rooms for adolescents to seek support, sameday ART initiation, & ongoing psychosocial support via 1-on-1 adherence counselling. | ND | Facility | Ongoing psychosocial support via 1-on-1 adherence counselling, consultation rooms and onsite training and mentoring of healthcare workers on the delivery of adolescent friendly services to increase access to services. | Linkage to HIV care increased from 63% to 98%. The provision of adolescent-friendly services at primary healthcare facilities is essential to improving the uptake of HIV treatment. |
| 30.Teasdale 2019† | Patients | Pregnant women attending ANC visits took self-interview surveys. The survey asked about women’s interactions with HCWs. Survey results were shared with HFS at monthly quality improvement sessions. | Baseline + repeated | Facility | Continuous quality improvement planning with routine feedback in order to improve Pt-provider relationships. | There was no significant difference between groups for retention in care. This type intervention may be useful in improving Pt-provider relationships. |
| 31. Wachira 2022i† | HCP | HCWs were trained on principles of communication, provider-Pt relationship dynamics & motivational interviewing as well as EPC packaging that included clinic scheduling and treatment dialog. | Baseline | Facility | Facilitated communication between providers and Pts to establish a relationship and better rapport. | There was no significant difference between groups for retention in car, but there was a significant positive effect from 64.4% to 84.4% |
| 31. Wachira 2022ii† | HCP | HCWs were trained on principles of communication, provider-Pt relationship dynamics & motivational interviewing. | Baseline | Facility | Facilitated communication between providers and Pts to establish a relationship and better rapport. | There was no significant difference between groups for retention in car, but there was a significant positive effect from 64.4% to 83.7% |
| 32. Zanoni 2017 | Clinic staff | Provide adolescent-friendly services outside of regular school time providing more time for Pt-provider relationships to develop. | Baseline + repeated | Facility | Provided adolescent-friendly services to eliminate barriers for adolescents and develop strong relationships with clinical staff. | The intervention had a significant positive effect on retention in care from 85% to 95.5% as well as viral suppression from 75.8% to 90%. |

Pts, Patients; FSW, Female Sex Worker; YA, Young Adults; Appts, Appointments; HCP, Health Care Providers; ND, Not Described; PLWH, People living with HIV; HCW, Health Care Worker; SRH, Sexual and Reproductive Health; HCFs, Health Care Facilities

†Intervention designed primarily to improve patient-provider interaction, HCW, Healthcare workers

## Table S5a: Risk of bias for included studies – RCT or cRCT

|  | Selection bias | | Performance bias | Detection bias | Attrition bias | Reporting bias | Cluster recruitment bias | Cluster baseline imbalance | Cluster loss of clusters | Cluster incorrect analysis | Cluster comparability | Overall Score |
| --- | --- | --- | --- | --- | --- | --- | --- | --- | --- | --- | --- | --- |
| Study | Random sequence generation | Allocation concealment | Blinding of participants and personnel | Blinding of outcome assessment | Incomplete outcome data | Selective reporting | Recruitment bias | Baseline imbalance | Loss of cluster | Incorrect analysis | Comparability with individually randomized trials |  |
| Hickey†, 2020 | Unclear | Unclear | Unclear | Unclear | Unclear | Low | Low | Unclear | Low | Low | Unclear | High |
| Mwangwa†, 2022 | Unclear | Unclear | Low | Low | Low | Low | High | Low | Low | Low | Unclear | Unclear |
| Jones, 2013 | Low | Unclear | High | Low | Low | Low | NA | NA | NA | NA | NA | High |
| Jones, 2016 | Low | Low | Unclear | Low | High | Unclear | NA | NA | NA | NA | NA | High |
| Kerrigan, 2019 | Low | Low | Unclear | Low | Low | Low | NA | NA | NA | NA | NA | Unclear |
| Wachira, 2022 | Low | Low | Low | Unclear | Low | Low | NA | NA | NA | NA | NA | Low |

†These studies are cRCTs

## Table S5b: Risk of bias for included studies – cohort studies and pre-post studies

|  | **Selection of cohort**  (maximum quality:4 starts) | | | | **Comparability**  (maximum quality:2 starts) | **Outcomes**  (maximum quality: 3 stars) | | | **Overall score** (max:9) |
| --- | --- | --- | --- | --- | --- | --- | --- | --- | --- |
| Study | Representativeness of the exposed cohort | Selection of the non-exposed | Ascertainment of exposure | Outcome of interest was not present at start of the study | Comparability of the design or analysis | Assessment of outcome | Follow up time sufficient | Adequacy of follow up cohorts |  |
| Ayieko, 2019 | * | - | * | * | ** | - | * | * | 7 |
| Balzer, 2017 | * | - | * | * | - | - | * | - | 4 |
| Jakubowski, 2022 | * | * | * | * | ** | * | * | - | 8 |
| Erb, 2017 | * | - | * | * | - | - | * | * | 5 |
| Keene, 2019 | - | - | - | - | - | - | - | - | 0 |
| Moucheraud, 2020 | * | * | * | * | - | * | * | * | 7 |
| Reif, 2019 | * | - | - | * | - | * | * | * | 5 |
| Onokala, 2015† | * | - | * | * | - | - | * | - | 4 |
| PATA, 2017† | - | - | * | * | - | - | * | - | 3 |
| Ruria, 2017† | * | - | * | * | - | * | * | - | 4 |
| Mburu, 2019† | * | - | * | * | ** | * | * | * | 8 |
| Teasdale, 2019† | - | * | * | * | - | * | - | - | 4 |
| Emerenini, 2019† | - | * | - | * | - | - | * | - | 3 |
| Smith, 2019† | - | - | * | * | - | * | * | - | 4 |
| Laterra 2020† | * | - | - | - | - | * | * | - | 3 |

†These studies are pre-post studies

The more stars a study has the higher the quality of the study (i.e. low risk of bias). This is based on the eight-question checklist from the Newcastle Ottawa Scale. Each section has a maximum number of stars that can be awarded shown above.

## Table S5c: Risk of bias for included studies – cross-sectional studies

|  | **Selection**  (maximum qualtiy:5 starts) | | | | **Comparability**  (maximum quality:2 stars) | **Outcomes** (maximum quality: 3 stars) | | **Overall Score** (max:10) |
| --- | --- | --- | --- | --- | --- | --- | --- | --- |
| Study | Representativeness of the sample | Sample size | Non-respondents | Ascertainment of the exposure (risk factor) | Comparability of the subjects in different outcome groups based on study design or analysis | Assessment of outcome | Statistical test |  |
| Galy, 2018 | * | - | - | * | - | * | * | 4 |

The more stars a study has the higher the quality of the study (i.e. low risk of bias). This is based on the eight-question checklist from the Newcastle Ottawa Scale. Each section has a max number of stars that can be awarded shown above.

## Table S5d: Risk of bias for included studies – qualitative studies

|  | **Question 1** | **Question 2** | **Question 3** | **Question 4** | **Question 5** | **Question 6** | **Question 7** | **Question 8** | **Question 9** | **Question 10** | **Overall Appraisal** |
| --- | --- | --- | --- | --- | --- | --- | --- | --- | --- | --- | --- |
| Study | Congruity v between research methodology | Congruity between methodology and research question | Congruity between research methods and data collection | Congruity between research methods and data analysis | Congruity between research methods and results | Researcher location culturally or theoretically | Influence on research from researcher addressed | Are participants voices represented | Ethical criteria and approval from appropriate body | Flow from analysis, interpretation and the data |  |
| Medina-Marino, 2020 | Unclear | Yes | Yes | Unclear | Yes | No | Unclear | Yes | Yes | Yes | include |
| Mutambo, 2020 | Unclear | Yes | Yes | Yes | Yes | No | Unclear | Yes | Yes | Yes | Include |

## Table S5e: Risk of bias for included studies – mixed methods studies

|  | **Qualitative** | | | | | **RCTs** | | | | | **Quantitative Non-randomized** | | | | | **Mixed Methods** | | | | | **Overall Score** |
| --- | --- | --- | --- | --- | --- | --- | --- | --- | --- | --- | --- | --- | --- | --- | --- | --- | --- | --- | --- | --- | --- |
| Study | Qualitative approach appropriate | Data collection methods adequate | Findings derived from the data | Interpretation of results with data | Coherence between all study parts | Randomized performed? | Comparability of groups at baseline | Complete outcome data | Outcome assessors blinded | Adhere to assigned intervention | Participants’ representative of pop. | Measurements appropriate | Complete outcome data | Confounders accounted for | Intervention administered correctly | Rational for using mixed methods | Components of study integrated | Components interpreted correctly | Inconsistencies addressed | Quality of criteria for each method |  |
| Kays, 2021 | * | 0 | * | 0 | * |  |  |  |  |  | * | * | * | 0 | * | * | * | 0 | 0 | * | Fair |
| Lowther, 2018 | * | * | * | * | * | * | * | * | 0 | 0 |  |  |  |  |  | * | * | * | 0 | * | Fair |
| Penfold, 2019 | * | 0 | 0 | 0 | * |  |  |  |  |  | * | 0 | * | 0 | 0 | * | * | 0 | 0 | 0 | Poor |
| PuttKammer, 2020 | * | * | * | * | * |  |  |  |  |  | * | * | 0 | 0 | * | * | * | * | 0 | * | Fair |
| Abboah-Offei, 2020 | * | 0 | * | * | * | * | * | * | 0 | * |  |  |  |  |  | * | 0 | * | 0 | * | Fair |
| Pascoe, 2019 | * | * | 0 | 0 | * | * | 0 | * | 0 | * |  |  |  |  |  | 0 | * | * | 0 | * | Poor |
| MacLachlan, 2016 | * | * | * | * | * | 0 | * | 0 | * | * |  |  |  |  |  | 0 | 0 | * | * | * | Fair |
| Zanoni, 2017 | * | * | * | * | * |  |  |  |  |  | * | * | * | * | * | 0 | 0 | 0 | * | * | Fair |
| Based on the Mixed Methods Appraisal Tool (MMAT), Studies designated as poor had a high risk of bias and those designated as fair had some risk of bias. 0= No or can’t tell for that question | | | | | | | | | | | | | | | | | | | | | |

## Table S6: Scholl domains by outcomes with the percent of study reports having a positive effect among study reports with a comparison arm

| **Scholl Domain** | **HCW-Pt relation** | **HCW-Pt Comm** | **Coord care** | **Access care** | **Pt info** | **Phys support** | **Emo support** | **Biopsych** | **Integration** | **Pt unique** | **HCW character** | **Teamwork** | **Pt involve** | **Fam involve** | **Pt empowerment** |
| --- | --- | --- | --- | --- | --- | --- | --- | --- | --- | --- | --- | --- | --- | --- | --- |
| **HIV Outcomes** | | | | | | | | | | | | | | | |
| Linkage | 100%  (n= 3) |  |  | 100%  (n= 1) |  |  | 100%  (n= 1) | 100%  (n= 3) | 100%  (n= 1) | 100%  (n= 1) | 100%  (n= 3) |  |  |  |  |
| ART initiation | 0%  (n= 2) |  |  | 0%  (n= 1) | 0%  (n= 1) |  |  | 0%  (n= 1) |  | 0%  (n= 1) | 0%  (n= 1) |  | 0%  (n= 1) |  |  |
| Adherence | 57%  (n= 7) | 40%  (n= 5) |  |  | 0%  (n= 2) |  |  | 100%  (n= 1) | 100%  (n= 1) | 100%  (n= 2) | 100%  (n= 1) | 100%  (n= 1) | 40%  (n= 5) |  | 67%  (n= 3) |
| Viral suppression | 57%  (n= 14) | 60%  (n= 10) | 100%  (n= 6) | 100%  (n= 8) | 67%  (n= 6) | 100%  (n= 3) | 100%  (n= 1) | 60%  (n= 5) | 50%  (n= 2) | 75%  (n= 4) | 50%  (n= 4) | 100%  (n= 2) | 33%  (n= 6) | 100%  (n= 1) | 60%  (n= 5) |
| Retention | 58%  (n= 12) | 44%  (n= 9) | 60%  (n= 5) | 71%  (n= 7) | 67%  (n= 6) | 100%  (n= 3) | 100%  (n= 1) | 67%  (n= 6) | 67%  (n= 3) | 40%  (n= 5) | 100%  (n= 2) | 0%  (n= 2) | 0%  (n= 5) |  | 50%  (n= 4) |
| **PCC Outcomes** | | | | | | | | | | | | | | | |
| Patient satisfaction | 100%  (n= 1) | 50%  (n= 2) |  |  | 100%  (n= 1) |  |  | 0%  (n= 1) |  |  |  |  | 50%  (n= 2) |  | 100%  (n= 1) |
| Patient-provider communication | 100%  (n= 2) | 100%  (n= 4) |  |  | 100%  (n= 2) |  |  | 100%  (n= 1) |  |  |  |  | 100%  (n= 4) |  | 100%  (n= 2) |
| Perceived Quality of Care |  | 100%  (n= 3) |  |  |  |  |  |  |  |  |  |  | 100%  (n= 3) |  |  |
| Other Identified Mechanisms | 33%  (n= 6) | 50%  (n= 6) |  | 67%  (n= 4) | 33%  (n= 6) |  | 67%  (n= 3) | 83%  (n= 6) |  |  |  | 67%  (n= 3) | 50%  (n= 6) |  | 0%  (n= 3) |
| **Economic Outcomes** | | | | | | | | | | | | | | | |
| Economic-related outcomes | 100%  (n= 4) | 100%  (n= 4) | 100%  (n= 4) | 100%  (n= 4) | 100%  (n= 4) | 100%  (n= 4) |  |  |  |  |  |  |  |  |  |

## Table S7: PCC intervention components; number of studies that have components

| **PCC Components** | **Friendly services** | **HCW feedback** | **HCW sensitisation** | **Imp indiv counselling** | **Pt empowerment** |
| --- | --- | --- | --- | --- | --- |
| **Friendly services** |  | 1 | 8 | 5 | 1 |
| **HCW feedback** | 1 |  | 1 | 0 | 0 |
| **HCW sensitisation** | 8 | 1 |  | 4 | 0 |
| **Imp indiv counseling** | 5 | 0 | 4 |  | 5 |
| **Pt training** | 1 | 0 | 0 | 5 |  |
